# Transfer Hydrogenation with Waste‐Derived Aqueous Solutions of Formic Acid Catalysed by the SulfoShvo Catalyst

**DOI:** 10.1002/cssc.202501832

**Published:** 2025-10-27

**Authors:** Justus Diekamp, Milan D. Kulaš, Jakob Albert, Thomas Seidensticker

**Affiliations:** ^1^ Department for Biochemical and Chemical Engineering Laboratory of Industrial Chemistry TU Dortmund University Emil‐Figge‐Straße 66 44227 Dortmund Germany; ^2^ Institute of Technical and Macromolecular Chemistry University of Hamburg Bundesstraße 45 20146 Hamburg Germany

**Keywords:** biomass, homogeneous catalysis, reduction, renewable resources, ruthenium

## Abstract

The OxFA process provides aqueous formic acid (FA) solutions of around 53 wt% directly from biomass‐derived byproducts such as glycerol. The direct utilisation of this diluted FA stream in transfer hydrogenation reactions without neutralisation or buffering is demonstrated, enabled by the highly hydrophilic, acid‐stable sulfoShvo catalyst operating at pH 1. Using levulinic acid (LA) as a model substrate, optimisation in pressure autoclaves identified 130 °C and catalyst loadings as low as 0.2 mol% as effective reaction conditions. Even under these harsh acidic conditions, the catalyst displays remarkable performance: at 100 °C, long‐term stability is achieved with a turnover number (TON) of 3680, while operation at 130 °C allows efficient hydrogenation but reveals gradual deactivation at very low loadings (0.025 mol%). Beyond LA, a range of water‐soluble carbonyl and olefinic substrates is successfully hydrogenated, highlighting the broad applicability of this sustainable approach. Overall, this study establishes the OxFA/sulfoShvo system as a robust and atom‐economical strategy for the direct use of biomass‐derived FA solutions in catalytic hydrogenation.

## Graphical abstract

Transfer hydrogenation of carbonyl and olefinic compounds with the buffer‐free combination of waste‐derived, aqueous formic acid solution and the sulfoShvo catalyst at pH = 1 and elevated temperatures in pressure autoclaves.

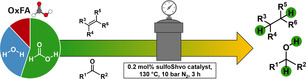

## 1 Introduction

The anticipated stepwise switch from fossil to regenerative feedstocks for the chemical industry entails an increased demand for reductive processes due to the high oxidation grade of bio‐based compounds. While the majority of these reductions will certainly be based on molecular hydrogen from electrolysis, the safety and storage disadvantages of dihydrogen, as well as a possible utilisation of waste and by‐product streams, leave a gap for the use of hydrogen carriers and surrogates.^[^ ^1^ ^]^ Transfer hydrogenation with such substitutes for gaseous hydrogen has been in the focus of research for several decades but has not been put to use in the chemical industry on a large scale until now.^[^ ^2^ ^]^ In 2012, Albert et al. first presented the catalytic oxidation of biomass to formic acid (FA) facilitated by Keggin‐type polyoxometalate (H_5_PV_2_Mo_10_O_40_) catalysts.^[^ ^3^ ^]^ This initial publication was followed by several in‐depth studies of the concept and the actual commercial application in the patented OxFA‐process.^[^ ^4^, ^5^, ^6^, ^7^, ^8^, ^9^, ^10^ ^]^ It was shown that all kinds of different carbon sources can be converted which makes the process highly interesting for the utilisation of waste streams. Today, the two main sources for the commercial process are the sugar hydrolysate Renmatix and raw glycerol from the production of biodiesel.^[^ ^11^, ^12^ ^]^ Considering, that formic acid is an ideal H_2_ surrogate, the usage of this biogenic hydrogen source for the reduction of biomass‐derived platform chemicals is highly favourable. The OxFA process yields a 50–60 wt% solution of crude formic acid in water with some minor organic residues (acetic acid, intermediates, and residues of substrate), which then needs to be concentrated for commercial use. The resource consumption of the water/impurity separation could be avoided if the solution yielded by the process could be directly brought to use.

**Figure 1:**
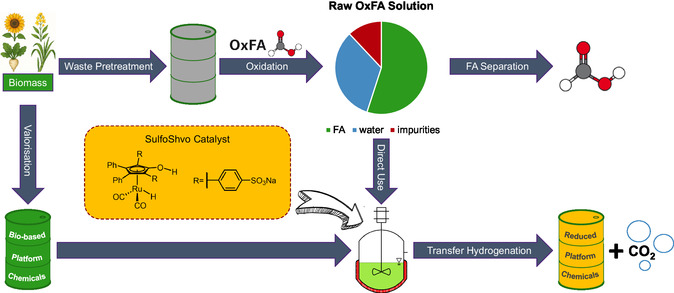
Flow scheme of the production of reduced, bio‐based platform chemicals through utilisation of the raw OxFA solution without an external hydrogen source as proposed in this publication.

Homogeneous transfer hydrogenation (TH) catalysts are an intensively researched field but most of them are not well suited for the application with aqueous solutions of formic acid. A majority is not water‐soluble/‐tolerant and those who are often do not tolerate the acidic environment and therefore use HCOONa solutions or buffered FA solutions.^[^ ^2^, ^13^ ^]^ We have recently developed a highly hydrophilic variant of Shvo's catalyst, sulfoShvo, which is perfectly soluble in aqueous solutions, non‐base‐activated, and whose ligand's diene donor moiety is not as easily protonated as heteroatoms. Both ligand and complex are easily synthesised in good to excellent yields.^[^ ^14^ ^]^ In this article, we want to demonstrate the potential that lies in the direct utilisation of the aqueous formic acid solution of the OxFA process by applying it together with our hydrophilic sulfoShvo catalyst (**Figure** **1**).

## 2 Results and Discussion

**Figure 2:**
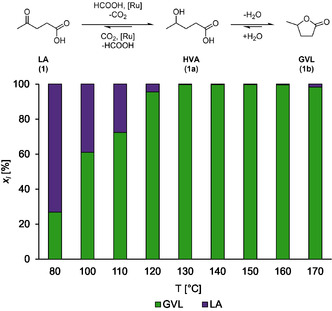
Results of the TH of levulinic acid with OxFA solution catalysed by the SulfoShvo catalyst. Reaction conditions: 3.9 mmol LA (1), 1.8 eq. FA, S/Ru = 100, 10 bar N_2_, 30 min.

The general capability of the sulfoShvo catalyst to transfer hydrogenate levulinic acid (LA) (**1**) in aqueous solutions of formic acid has been proven in our initial publication on this catalyst system.^[^ ^14^ ^]^ This reaction has been found to be an ideal model since both substrate and product are highly water‐soluble and the fast cyclisation of the hydrogenation product 4‐hydroxy valeric acid (**1a**) to *γ*‐valero lactone (GVL) (**1b**) under acidic conditions at elevated temperatures simplifies the analytics of the reaction by avoiding potential formates of the product alcohol (**Figure** **2**). Considering literature and our insights regarding the reaction temperature for TH reactions with Shvo's catalyst and the sulfoShvo catalyst (>70 °C), the optimisation window is rather small when the boiling points of water (100 °C) and FA (101 °C) are the upper limit in an open reflux set‐up.^[^ ^14^, ^15^, ^16^ ^]^ Consequently, a pressure‐stable closed system seemed beneficial. We chose to operate our reactions in stainless steel pressure autoclaves. To limit the contact between the autoclave and the corrosive, hot FA, the reaction mixtures were placed in glass vials whose septa were pierced by small PTFE cannulas to allow pressure equalisation. Boiling delay was avoided by pressurising the autoclaves with 10 bar N_2_ prior to the reaction.

The temperature screening covered the range from 70 up to 170 °C (limited by the autoclaves) and was done with an initial catalyst load of 1 mol%. It quickly became obvious that the catalyst load could be reduced since all reactions between 130 and 160 °C reached full conversion after 30 min (Figure 2). The reaction at 170 °C only reached 98% GVL (**1b**) yield and showed black Ru particles (composition verified via ICP‐OES) after the reaction, indicating the decomposition of the catalyst at high temperatures. Black particles were also found in the reactions run at 150 °C and 160 °C.

**Figure 3:**
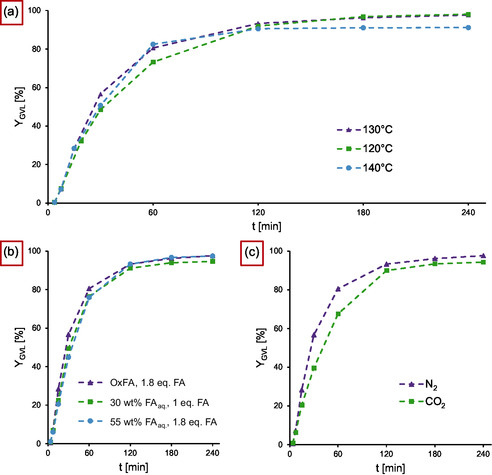
GVL (1b) yields over time for different reaction conditions. a) Comparison of reaction temperatures. Reaction conditions: 19 mmol LA (1), 1.8 eq. FA, S/Ru = 500, 10 bar N_2_. b) Comparison of different OxFA solutions and artificial FA/H_2_O mixtures. Reaction conditions: 19 mmol LA (1), 1.8/1.1 eq. FA, S/Ru = 500, 130 °C, 10 bar N_2_. c) Investigation of the influence of CO_2_ concentration. Reaction conditions: 19 mmol LA (1), 1.8 eq. FA, S/Ru = 500, 130 °C, 10 bar.

To determine differences in reaction rate and a possible reduction of the catalyst load, we performed a series of experiments with 0.2 mol% catalyst. While the reaction profiles of 120 °C and 130 °C showed only minor differences in the beginning, the reaction at 140 °C only reached 91% GVL (**1b**) after 4 h compared to 98% for 120 °C and 130 °C (**Figure** **3**a). Again, the formation of black particles was observed. Overall, the temperature dependence of the yield curves in the investigated temperature window is comparatively small. This can be attributed to increasing rates of catalyst deactivation, which counter an increased reduction rate. Since the glycerol‐derived OxFA solution, which we utilised, contains residues of glycerol and various intermediates of the process (see Supporting Information), it was benchmarked against a 55 wt% solution of formic acid in water to investigate a possible negative impact of such impurities (Figure 3b). Both reactions reached 97% GVL (**1b**) yield after 4 h, demonstrating the feasibility of OxFA solutions as a hydrogen source. A reduction of the FA equivalents to 1 showed a similar reaction rate but yielded 95% GVL (**1b**) after 4 h (Figure 3b), which might be the consequence of small amounts of unproductive decomposition of FA to CO_2_ and H_2_. The fact that all three FA solutions produce comparable results indicates an insensitivity to FA concentrations. A possible explanation could be that the hydrogen abstraction from FA by the complex is significantly faster than the reduction of the carbonyl moiety, thereby creating a kinetic bottleneck that is independent of the FA concentration. To investigate the effect of a possible competing CO_2_ reduction on the GVL (**1b**) yield in the closed set‐up, a reaction was run in the same fashion as before, but with 10 bar CO_2_ instead of N_2_. The result was a reduced reaction rate but still a yield of 94% GVL (**1b**) after 4 h (Figure 3c).

**Figure 4:**
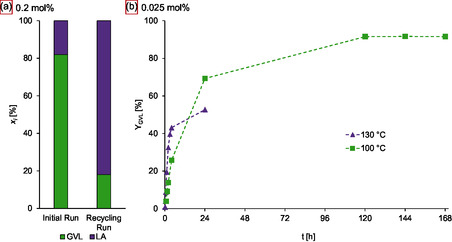
a) Results of the initial run and the recycling run after 1 h reaction time and b) deactivation experiments with low catalyst load of the sulfoShvo catalyst in the model TH of levulinic acid (1) with OxFA solution. Reaction conditions: a) 19 mmol LA (1), 1.8 eq. FA, S/Ru = 500, 130 °C, 10 bar N_2_, 1 h. b) 19 mmol LA (1), 1.8 eq. FA, S/Ru = 4000, 10 bar N_2_.

In order to assess the stability of the catalyst system, a TH reaction of levulinic acid (**1**) with 0.2 mol% catalyst was run for 4 h up to full conversion, with a sample drawn after 1 h. Afterwards, the volatile compounds of the reaction mixture were removed in vacuo, the remaining catalyst slurry was recharged with glycerol‐derived OxFA solution and levulinic acid (**1**), and the reaction run a second time. **Figure** **4**a shows a significant drop in yield from the initial run (82% at 1 h) to the recycling run (18% at 1 h), indicating catalyst deactivation. Further investigations of the reaction profiles at 130 °C with a low catalyst load of only 0.025 mol% showed 53% GVL (**1b**) yield after 24 h (Figure 4a) and an early decrease in reaction rate compared to the reaction with 0.2 mol%. Lowering the reaction temperature to 100 °C resulted in an initially slower reaction as expected, but much‐improved long‐term activity, reaching 69% of GVL (**1b**) yield after 24 h and a plateau of 92% after 5 days. This translates into a TON of 3680 and shows a remarkable catalyst stability under the harsh conditions of the reaction. Contrary to the reactions at T ≥140 °C from the initial reaction temperature screening, the solutions of the 0.025 mol% reactions as well as the recycling experiment remained clear yellow/orange solutions without black particles. Hence, a different deactivation pathway than simple thermal decomposition may lead to reduced catalyst activity. Van Slagmaat et al. have studied the deactivation of Shvo's catalyst in the presence of water but proposed a stabilising effect of formic acid adducts due to a conflicting study by Fábos et al.^[^ ^15^, ^17^ ^]^ It was suggested that the deactivation of the catalyst proceeds through the deoxygenation of the non‐innocent carbonyl moiety,^[^ ^17^ ^]^ which would eliminate the ligand's capability to transfer the proton but maintain a ruthenium complex. A separate deactivation experiment with the sulfoShvo catalyst in aqueous FA solution revealed changes in the ^13^C NMR spectrum but did not match the spectra of van Slagmaat et al. New signals between 8 ppm and 29 ppm after the reaction indicate the formation of alkyl groups, which hints toward catalyst degradation through reduction of the cyclopentadienone ligand. While the NMR spectra clearly show a change in the structure of the catalyst, the exact pathway and products remain elusive and need further investigation.

**Figure 5:**
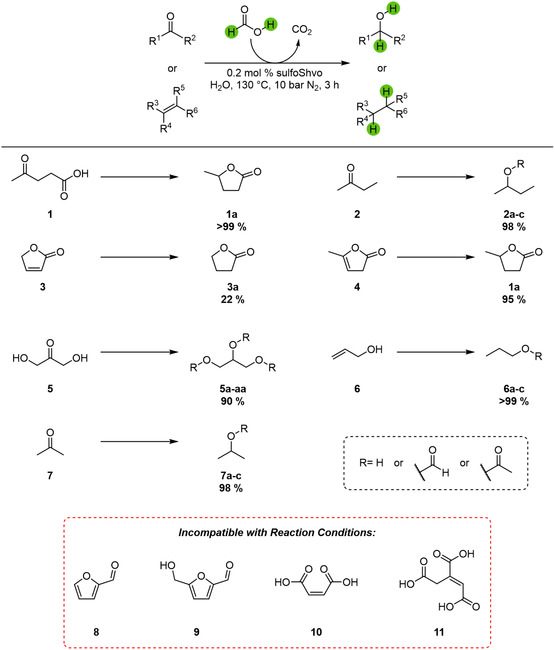
Yields of the TH of various water‐soluble carbonyl and olefin compounds in OxFA solution catalysed by the sulfoShvo catalyst. Reaction conditions: 3.9 mmol substrate, 620 mg OxFA solution (1.8 eq. FA), S/Ru = 500, 130 °C, 10 bar N_2_, 3 h.

**Scheme 1:**
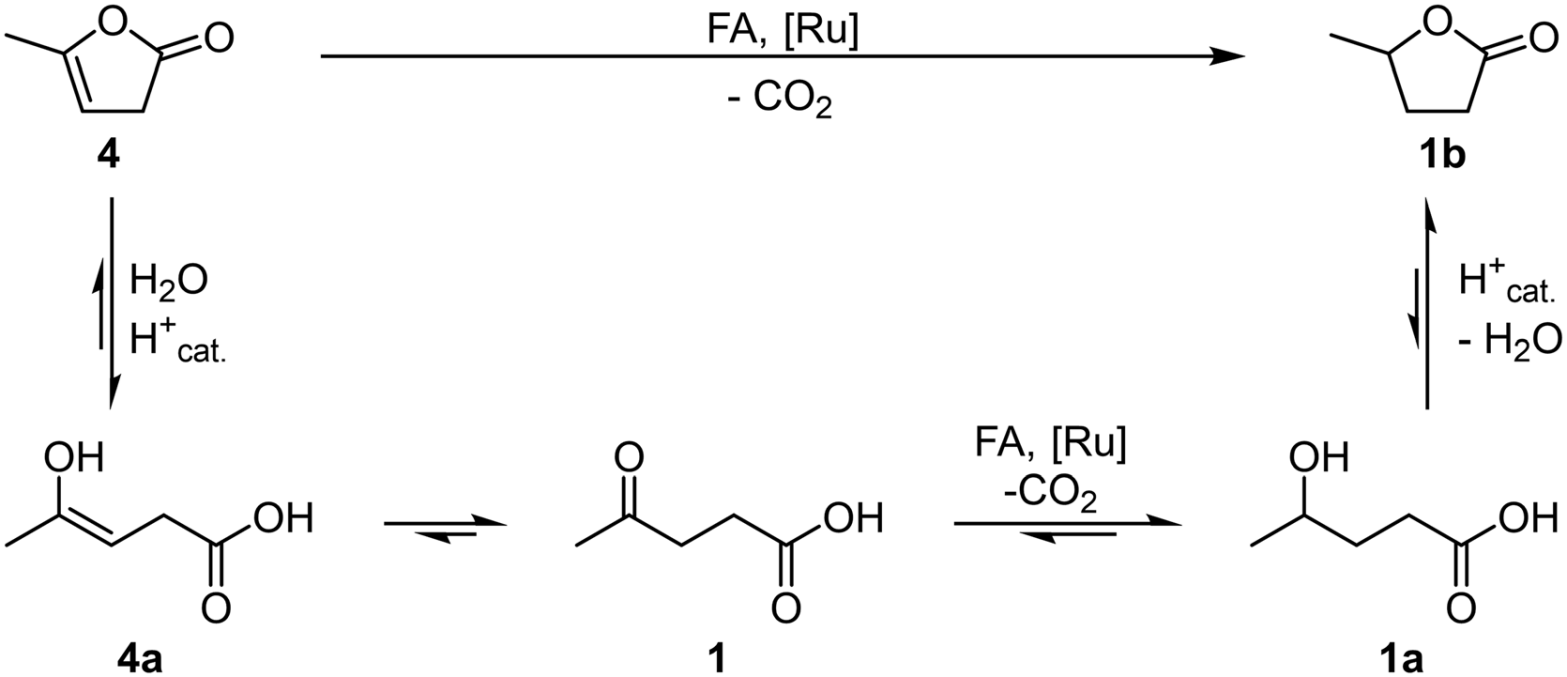
Direct reduction of α‐angelica lactone to *γ*‐valero lactone catalysed by the sulfoShvo catalyst versus the indirect reduction via the hydrolysis/isomerizsation/reduction/lactonisation cascade.

The substrate scope covered a variety of water‐soluble carbonyl and olefin compounds with a focus on biomass‐derived substances (**Figure** **5**). Several substrates were successfully transfer hydrogenated. The carbonyl compounds butanone (**2**), dihydroxyacetone (**5**), and acetone (**7**) showed excellent yields. Since their products are alcohols, the given yields include the corresponding esters with formic acid and acetic acid, which formed subsequently to the reduction. It is important to note that the missing 10% in the TH of dihydroxyacetone (**5**) are not the remaining substrate but the side‐product 1,3‐propanediol (**5ab**), in which the carbonyl moiety was also reduced. The investigated olefinic compounds were 2(5*H*)‐furanone (**3**), α‐angelica lactone (**4**), and allyl alcohol (**6**), the latter yielding alcohol and esters as well. While allyl alcohol (**6**) and α‐angelica lactone (**4**) were converted in full, 2(5*H*)‐furanone (**3**) yielded only 22% of butyrolactone (**3a**). It is important to note that there are two pathways from α‐angelica lactone (**4**) to its hydrogenation product GVL (**1b**). Apart from direct hydrogenation, it is possible that the lactone (**4**) first undergoes an acid‐catalysed hydrolysis with subsequent isomerisation to levulinic acid (**1**), which is then transfer hydrogenated and yields GVL (**1b**) after lactonisation (**Scheme** **1**).

This route is supported by the fact that 5% of levulinic acid have been found in the reaction mixture after 3 h. Furthermore, the low conversion of 2(5*H*)‐furanone (**3**) raises the question if the direct reduction of the C=C double bond is fast enough to reach such high yields in the TH of α‐angelica lactone (**4**). Three standard bio‐based platform chemicals we investigated were furfural (**8**), 5‐HMF (**9**), and maleic acid (**10**). All three were not successfully converted due to the highly acidic reaction conditions. Furfural (**8**) and 5‐HMF (**9**) polymerised to dark brown humins, while maleic acid (**10**) was isomerised through acid catalysis to the insoluble fumaric acid and precipitated.^[^ ^18^ ^]^ A similar problem was observed for *trans*‐aconitic acid (**11**), which decarboxylated to the insoluble itaconic acid at reaction conditions.

## 3 Conclusion

An aqueous solution of formic acid (53 wt%) derived from wet biomass via the OxFA process was successfully combined with the readily water‐soluble sulfoShvo catalyst to transfer hydrogenate levulinic acid at elevated temperatures in a pressure autoclave. The optimisation of the reaction conditions with levulinic acid (LA) as the model substrate led to a reduction of the catalyst load down to 0.2 mol% and a reaction temperature of 130 °C. The attempted recycling of the catalyst and the further reduction of the catalyst load down to 0.025 mol% revealed catalyst deactivation at 130 °C, but at the same time demonstrated long‐term catalyst stability and an excellent TON of 3680 at 100 °C despite the harsh conditions. Furthermore, a variety of water‐soluble carbonyl and olefinic substrates were successfully transfer hydrogenated.^[^ ^19^ ^]^

## Conflict of Interest

The authors declare no conflict of interest.

## Supporting information

Supplementary Material

## Data Availability

The data that support the findings of this study are available from the corresponding author upon reasonable request.
