# Supplementary material for: Transfer Hydrogenation with Waste‐Derived Aqueous Solutions of Formic Acid Catalysed by the SulfoShvo Catalyst
Source: ChemSusChem. 2025 Oct 27;18(24):e202501832. doi: 10.1002/cssc.202501832 (PMC12703433; doi:10.1002/cssc.202501832)
Supplement: Supplementary file 1 — Supplementary Material [file CSSC-18-e202501832-s001.pdf]

Supporting Information

Transfer Hydrogenation with Waste-Derived Aqueous Solution of Formic Acid Catalysed by the SulfoShvo Catalyst

Authors: Justus Diekamp[a], Milan D. Kulaš[a], Jakob Albert[b], and Thomas Seidensticker*[a]

[a] TU Dortmund University, Department for Biochemical and Chemical Engineering, Laboratory of Industrial Chemistry, Emil-Figge-Straße 66, 44227 Dortmund, Germany

[b] Institute of Technical and Macromolecular Chemistry, University of Hamburg, Bundesstraße 45, Hamburg 20146, Germany.

*Dr. Thomas Seidensticker, email: thomas.seidensticker@tu-dortmund.de, phone: +49 231 755 2310

Table of Contents

Supporting Information 1

Table of Contents 1

1. Reagents 3

2. Ligand and Complex Synthesis 4

2.1. Ligand Synthesis 4

2.2. Complex Synthesis 5

3. General Procedures 6

3.1. Transfer Hydrogenation of Levulinic Acid (1) with OxFA Solution in Pressure Autoclaves 6

3.2. Transfer Hydrogenation of Levulinic Acid (1) in a Pressure Autoclave with a Sampling System 7

3.3. Transfer Hydrogenation of Liquid Water-Soluble Substrates in Pressure Autoclaves 7

3.4. Transfer Hydrogenation of Solid Water-Soluble Substrates in Pressure Autoclaves 8

4. Analytics 9

4.1. GC-TCD 10

4.2. NMR Analytics 12

4.2.1. NMR Spectra Catalyst Synthesis 13

4.2.2. Transfer Hydrogenation of 2-Butanone (2) 16

4.2.3. Transfer Hydrogenation of 2(5H)-furanone (3) 19

4.2.4. Transfer Hydrogenation of α-Angelica Lactone (4) 21

4.2.5. Transfer Hydrogenation of Dihydroxyacetone (5) 23

4.2.6. Transfer Hydrogenation of Allyl Alcohol (6) 25

4.2.7. Transfer Hydrogenation of Acetone (7) 27

4.3. ICP-OES 29

5. References 29

References .... **Error! Bookmark not defined.**

1. Reagents

All liquid chemicals were degassed with argon dispersed by a frit for one hour in an ultrasonic bath or *via* the freeze-pump-thaw method.

Table S 1: Chemicals used in this work.

Chemical	Number	Manufacturer	Purity [w%]
tetraphenylcyclopentadienone	**12**	Sigma Aldrich	98
trimethylsilyl chlorodisulfonate	**13**	Sigma Aldrich	99
$Ru_3(CO)_{12}$	**14**	Sigma Aldrich	99
methanol	**15**	Thermo Scientific	>99
1,2-dichlorethane	**16**	Carl Roth	99
ethanol	**17**	Carl Roth	99
sodium hydroxide	**18**	Thermo Scientific	>99
n-heptane	**19**	Thermo Scientific	>99
OxFA solution (53 wt% FA, from glycerol)		OxFA	/
formic acid	**20**	Thermo Scientific	98
levulinic acid (LA)	**1**	BLDpharm	97
γ-valero lactone (GVL)	**1b**	Thermo Scientific	>99
2-butanone	**2**	Thermo Scientific	99
2(5H)-furanone	**3**	Thermo Scientific	95
α-angelica lactone	**4**	Thermo Scientific	98
dihydroxyacetone	**5**	BLDpharm	98
allyl alcohol	**6**	Sigma Aldrich	98
acetone	**7**	Carl Roth	>99
furfural	**8**	abcr	98
5-hydroxymethylfurfural	**9**	abcr	98
maleic acid	**10**	Thermo Scientific	98
aconitic acid	**11**	abcr	98
acetic acid	**21**	Carl Roth	>99
Argon		Messer SE	99.996
N_2		Messer SE	99.5
CO_2		Messer SE	99.996

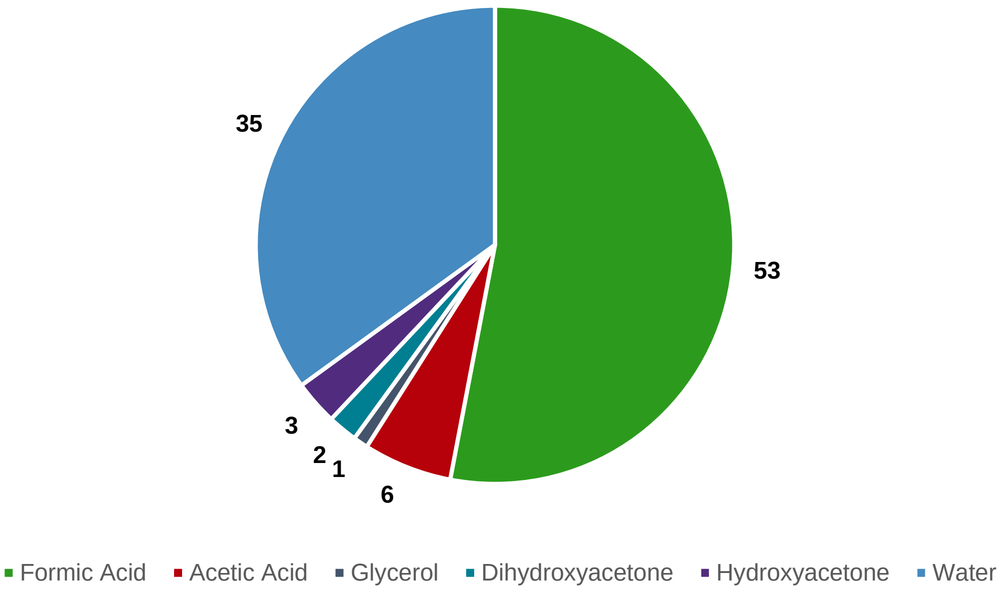

Figure S 1: Composition (in wt%) of the OxFA solution (53 wt% FA) derived from raw glycerol.

2. Ligand and Complex Synthesis

2.1. Ligand Synthesis

4,4'-(2-oxo-4,5-diphenylcyclopenta-3,5-diene-1,3-diyl)dibenzenesulfonic acid (22)

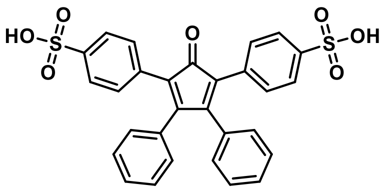

The disulfonic acid was synthesised accordingly to the procedure reported by Skalski et al..[19] Our workup deviated from their procedure. After the quenching, the solution was concentrated and stored at 6 °C. The product crystallized, was filtered off and washed with cold heptane. The resulting dark violet solid (Y = 81 %), was dried in a vacuum oven at 100 °C. NMR studies revealed residues of solvent, which were not removed since the following neutralisation yielded a pure product without purification of the disulfonic acid.

^{1}H NMR (400 MHz, DMSO-d_6): δ = 8.76 (br, 2H), 7.47 (d, J = 7.8 Hz, 4H), 7.25 (tt, J = 8.8, 4.8 Hz, 6H), 7.12 (d, J = 7.9 Hz, 4H), 6.97 (d, J = 6.7 Hz, 4H) ppm.

^{13}C {^{1}H} NMR (151 MHz, DMSO-d_6) δ = 199.5, 155.0, 147.1, 132.6, 130.7, 129.2, 128.9, 128.7, 128.1, 125.2, 124.6, 39.5 ppm.

HR-MS (ESI): calcd. $[M-2H]^{2-}$ = 271.0252, found $[M-2H]^{2-}$ = 271.0244.

Disodium 4,4'-(2-oxo-4,5-diphenylcyclopenta-3,5-diene-1,3-diyl)dibenzenesulfonate (23)

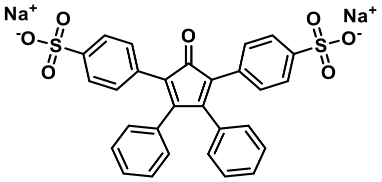

The disodium salt **23** was synthesised according to the procedure reported by Diekamp et al..[14] 2 g of the disulfonic acid **22** (5.2 mmol, 1 eq.) were dissolved 50 mL EtOH. 520 mg of NaOH (13 mmol, 2.5 eq.) were dissolved in 100 mL EtOH. The NaOH solution was added to the solution of **22** under constant stirring. The product precipitated instantly. The solid was filtered off, washed with EtOH, and dried in a vacuum oven at 110 °C yielding a grey-violet powder (Y = 90 %).

^{1}H NMR (400 MHz, DMSO-d_6): δ = 7.48 (d, *J* = 8.4 Hz, 4H), 7.32 – 7.20 (m, 7H), 7.12 (d, *J* = 8.4 Hz, 4H), 6.97 (dd, *J* = 8.0, 1.6 Hz, 4H) ppm.

^{13}C {^{1}H} NMR (101 MHz, DMSO-d_6): δ = 199.5, 155.0, 147.1, 132.6, 130.6, 129.2, 128.9, 128.7, 128.1, 125.2, 124.6 ppm.

HR-MS (ESI): calcd. $[M-2Na]^{2-}$ = 271.0252, found $[M-2Na]^{2-}$ = 271.0249.

2.2. Complex Synthesis

The sulfoShvo catalyst (**C1**) was synthesised according to the procedure reported by Diekamp et al..[14] A 500 mL Schlenk flask was charged with a stirring bar, 589 mg of the disulfonated ligand **23** (1 mmol, 3 eq.), and 213 mg of $Ru_3(CO)_{12}$ (**14**) (0.33 mmol, 1 eq.). 250 mL of dry methanol were added and the resulting mixture was refluxed for 40 h. Afterwards, the solvent was removed in vacuo yielding an orange solid which was washed with diethyl ether and dryed under high vacuum.

The structure of the isolated solid is not consistent with the results of the NMR measurements and the HR-MS spectrum due to the formation of the monomeric hydride complex, which was observed *via* NMR. Therefore all NMR and HR-MS measurements refer to that structure found in solution (Figure S2). For the NMR spectrum, the measurement after 20.5 h in solution (DMSO-d_6) was analysed. The undefined solid will be referred to as compound **C1**.

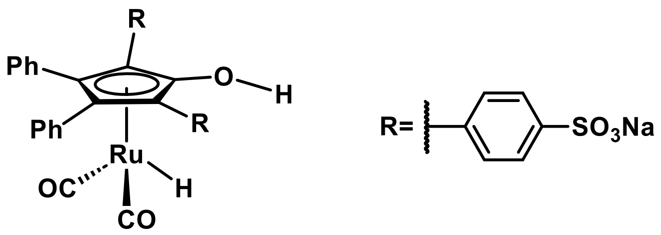

Figure S 2: Structure of the monomeric hydride complex, which forms in solution *via* dehydrogenation of MeOH.

^{1}H NMR (600 MHz, DMSO-d_6): δ = 10.10 (s, 2H, formaldehyde), 7.50 – 7.01 (m, 18H, CHaryl), 3.33 (s, 1H, OH), 3.17 (d, J = 5.1 Hz, 3H, CH3),-9.83 (s,1H, RuH) ppm.

^{13}C {^{1}H} NMR (151 MHz, DMSO-d6): δ = 202.1, 194.7, 173.5, 147.1, 146.8, 136.8, 132.6, 132.3, 131.9, 131.4, 131.1, 130.9, 129.7, 129.7, 128.5, 128.0, 127.6, 127.4, 125.0, 125.0, 124.2, 107.8, 104.0, 91.0, 80.2, 64.9, 48.6, 40.1, 39.9, 39.8, 39.7, 39.5, 39.4, 39.2, 39.1, 15.2 ppm.

HR-MS (ESI): calcd. [M-2Na]2- = 350.9805, found [M-2Na]2- = 350.9782.

Since methanol co-crystallises and all possible complex species can form the active monomeric hydride in solution in the presence of a hydrogen source[2], the catalyst load in the TH reactions was calculated based on the Ru content of the orange solid **C1**, which was determined *via* ICP-OES.

3. General Procedures

The heat transfer between heating block and the solution was tested for all reactor setups by filling the glass vials with silicon oil and placing the open reactor with the vial in a preheated heating block. The temperature of the silicon oil was monitored with a thermometer. The silicon oil reached the temperature of the heating block in under 8 min for every setup.

3.1. Transfer Hydrogenation of Levulinic Acid (1) with OxFA Solution in Pressure Autoclaves

A 9 mL glass vial was equipped with a 10x10x5 mm cross stirring bar and transferred into a glovebox. 29.6 mg of SulfoShvo catalyst (0.039 mmol, 1 mol%) was weighed into the vial and the vial was closed with a septum cap. The closed vial was removed from the glovebox and subsequently charged with 452 mg (3.9 mmol, 1 eq.) degassed levulinic acid (**1**) and 620 mg degassed OxFA solution (1.8 eq. FA) *via* syringe. A 10 mL stainless steel pressure autoclave was flushed with argon. The septum of the vial was pierced with a 1 mm stainless steel canula, a smaller piece of PTFE canula was inserted into the steel canula, and the steel canula was removed while the PTFE canula remained in the septum. Then the vial was quickly placed in

the flushed autoclave, which was then again closed with a constant argon flow running through the autoclave head. After closing, the autoclave was pressurised with 10 bar of nitrogen. Afterwards, the autoclave was placed in a preheated aluminium block at the desired reaction temperature on a combined heating/stirring plate. The magnetic stirrer was set to 600 rpm. After 30 min the autoclave was put into an ice bath, cooled down to room temperature, and depressurized. The vial was taken out and GC samples of the reaction mixture were prepared.

3.2. Transfer Hydrogenation of Levulinic Acid (1) in a Pressure Autoclave with a Sampling System

A 30 mL glass vial was equipped with a 10x10x5 mm cross stirring bar and transferred into a glovebox. SulfoShvo catalyst was weighed into the vial, and the vial was closed with a septum cap. The closed vial was removed from the glovebox and subsequently charged with 2.211 g (19 mmol, 1 eq.) degassed levulinic acid (**1**) and degassed FA solution (OxFA or 30 wt% FA in H_2O or 55 wt% FA in H_2O) *via* syringe. A 75 mL stainless steel pressure autoclave with a PTFE canula/valve combination for sampling was flushed with argon. The septum of the vial was pierced with the PTFE canula under constant argon flow. The septum of the vial was pierced with a 1 mm stainless steel canula, a smaller piece of PTFE canula was inserted into the steel canula, and the steel canula was removed while the piece of PTFE canula remained in the septum. Then the vial was quickly placed in the flushed autoclave, which was then again closed with a constant argon flow running through the autoclave head. After closing, the autoclave was pressurised with 10 bar of nitrogen or CO_2. Afterwards, the autoclave was placed in a preheated aluminium block (reaction temperature + 20 °C) on a combined heating/stirring plate. The magnetic stirrer was set to 600 rpm and the temperature to the reaction temperature. Samples were drawn *via* the PTFE canula with the valve.

3.3. Transfer Hydrogenation of Liquid Water-Soluble Substrates in Pressure Autoclaves

A 9 mL glass vial was equipped with a 10x10x5 mm cross stirring bar and transferred into a glovebox. 5.9 mg of SulfoShvo catalyst (0.0078 mmol, 0.2 mol%) was weighed into the vial and the vial was closed with a septum cap. The closed vial was removed from the glovebox and subsequently charged with 3.9 mmol of degassed substrate and 620 mg degassed OxFA solution (1.8 eq. FA) *via* syringe. A 10 mL stainless steel pressure autoclave was flushed with argon. The septum of the vial was pierced with a 1 mm stainless steel canula, a smaller piece of PTFE canula was inserted into the steel canula, and the steel canula was removed while the PTFE canula remained in the septum. Then the vial was quickly placed in the flushed autoclave, which was then again closed with a constant argon flow running through the autoclave head. After closing, the autoclave was pressurised with 10 bar of nitrogen. Afterwards, the autoclave was placed in a preheated aluminium block at 130 °C on a combined

heating/stirring plate. The magnetic stirrer was set to 600 rpm. After 3 h the autoclave was put into an ice bath, cooled down to room temperature, and depressurized. The vial was taken out and GC samples of the reaction mixture were prepared.

3.4. Transfer Hydrogenation of Solid Water-Soluble Substrates in Pressure Autoclaves

3.9 mmol of substrate were weighed into a 5 mL schlenk flask, which was then flushed with argon. The solid was dissolved in 1 mL of degassed water. A 9 mL glass vial was equipped with a 10x10x5 mm cross stirring bar and transferred into a glovebox. 5.9 mg of SulfoShvo catalyst (0.0078 mmol, 0.2 mol%) was weighed into the vial and the vial was closed with a septum cap. The closed vial was removed from the glovebox and subsequently charged with the substrate solution and 620 mg degassed OxFA solution (1.8 eq. FA) *via* syringe. A 10 mL stainless steel pressure autoclave was flushed with argon. The septum of the vial was pierced with a 1 mm stainless steel canula, a smaller piece of PTFE canula was inserted into the steel canula, and the steel canula was removed while the PTFE canula remained in the septum. Then the vial was quickly placed in the flushed autoclave, which was then again closed with a constant argon flow running through the autoclave head. After closing, the autoclave was pressurised with 10 bar of nitrogen. Afterwards, the autoclave was placed in a preheated aluminium block at 130 °C on a combined heating/stirring plate. The magnetic stirrer was set to 600 rpm. After 3 h the autoclave was put into an ice bath, cooled down to room temperature, and depressurized. The vial was taken out and GC samples of the reaction mixture were prepared.

3.5. Catalyst Deactivation Experiment

A 30 mL glass vial was equipped with a 10x10x5 mm cross stirring bar and transferred into a glovebox. 40 mg of sulfoShvo catalyst was weighed into the vial, and the vial was closed with a septum cap. The closed vial was removed from the glovebox and subsequently charged with 10 mL degassed FA solution (55 wt% FA in H_2O) *via* syringe. A 75 mL stainless steel pressure autoclave with a PTFE canula/valve combination for sampling was flushed with argon. The septum of the vial was pierced with the PTFE canula under constant argon flow. The septum of the vial was pierced with a 1 mm stainless steel canula, a smaller piece of PTFE canula was inserted into the steel canula, and the steel canula was removed while the piece of PTFE canula remained in the septum. Then the vial was quickly placed in the flushed autoclave, which was then again closed with a constant argon flow running through the autoclave head. After closing, the autoclave was pressurised with 10 bar of N_2. Afterwards, the autoclave was placed in a preheated aluminium block (120 °C) on a combined heating/stirring plate. The magnetic stirrer was set to 600 rpm. The reaction mixture was heated for 4 h and then cooled down to room temperature. The reactor was depressurised and the glass vial was placed into

a 200 mL Schlenk tube. The volatile components of the mixture were removed *in vacuo* and the solid residue was dissolved in deuterium oxide for NMR analysis.

4. Analytics

4.1. GC-TCD of the Gas Phase

Gas phase analysis was performed using an Agilent Technologies 7809A GC modified to mirror the setup described by Agilent in their application note 228-387. [75]

The setup makes the addition of a DB-1 column (10 m x 530 µm, x 5 µm), which is used to protect both the HP-PLOT Q (30 m x 530 µm, x 40 µm) as well as the HP-PLOT MOLESIEVE (30 m x 530 µm, x 50 µm) from solvents and reagents trapped in the gas phase. The HP-PLOT Q column protects the HP-PLOT MOLESIEVE from CO_2, and switching is achieved by a 6-way valve. Detection is achieved with two TCDs, one heated to 275°C and one to 200°C. The valve switches positions at 0 min, 1 min, 2.4 min, and 3.15 min, allowing the separate collection and measurement of water and carbon dioxide.

Table S 2: Heating profiles for the analysis of gas phases using GC-TCD.

	Rate [°C min^{-1}]	Temperature [°C]	Holding time [min]
T_{Start}		40	10

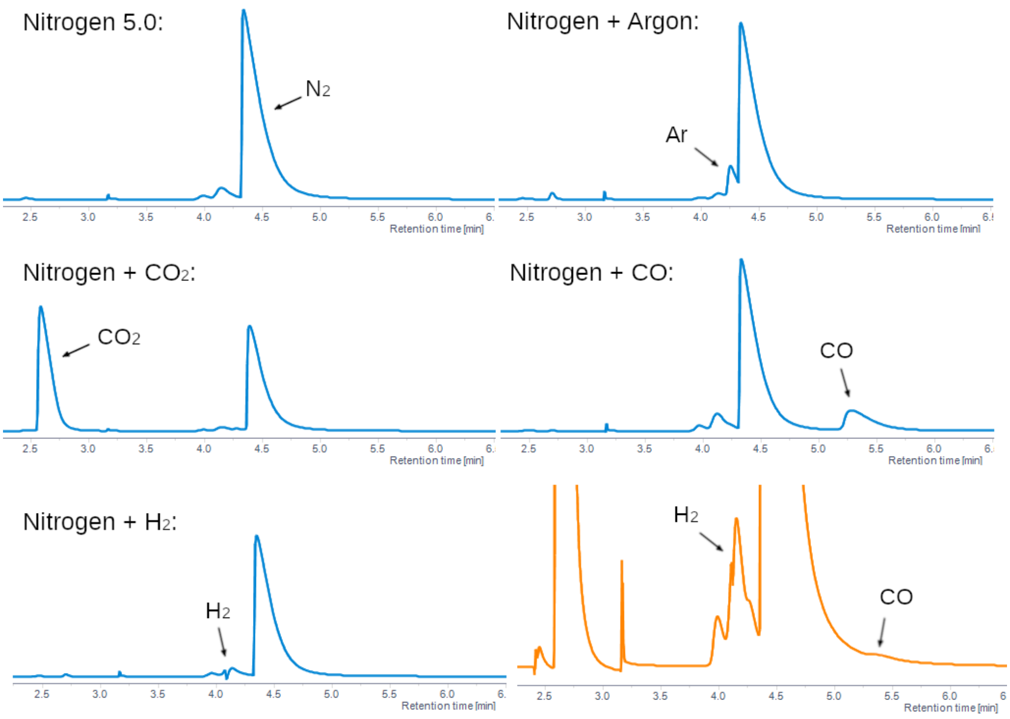

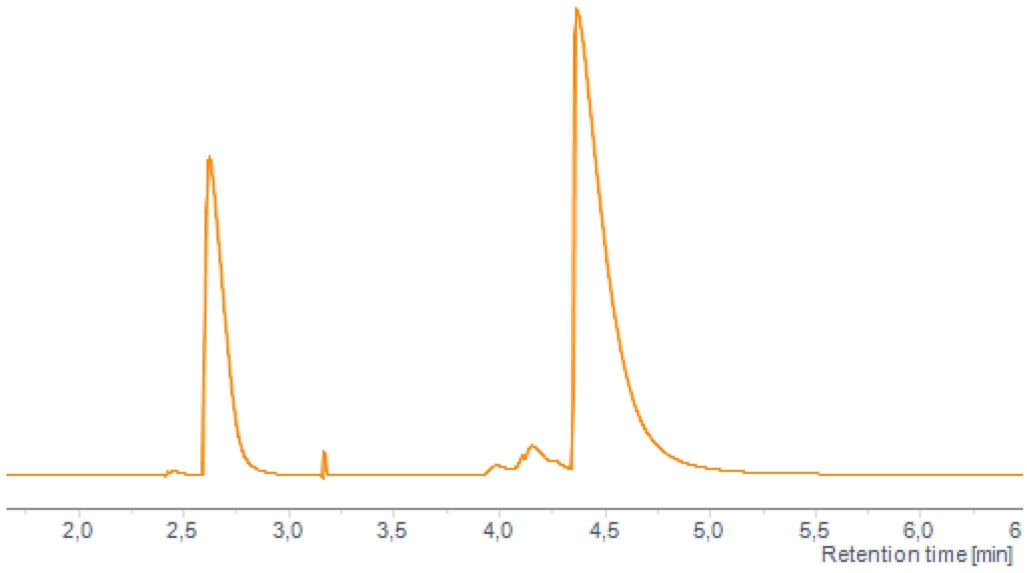

Figure S 3. _Blue traces from top left to bottom left, left to right:_ 15 bar nitrogen, 14 atm nitrogen with apox. 1 atm of argon, 10 atm nitrogen with 5 atm carbon dioxide, 10 atm nitrogen with 5 atm hydrogen, 14 atm nitrogen with aprox. 1 atm carbon monoxide; _Orange trace:_ Gas phase of the reaction of OxFA with 0.025 mol% sulfoShvo catalyst after 48 hours at 120 °C.

The composition of some post-reaction gas phases was analysed to determine if undesired decomposition of FA to either H_2 and CO_2 or H_2O and CO took place in the reactions. The setup used is capable of separating permanent gases, with carbon dioxide, nitrogen, argon, hydrogen, and carbon monoxide showing distinct and known retention times. For one sample (a reaction of 0.025 mol% after 48 hours at 120 °C), both hydrogen (negative peak at 4.1 min) and minute traces of carbon monoxide (5.4 min) could be confirmed (Figure **S 3**).

The observed amount, however, was far outweighed by the amount of carbon dioxide measured. Other samples tested (1 mol% and 0.2 mol% after 30 minutes at 110 °C) showed no trace of either gas.

4.2. GC-TCD

All measurements were performed using an Agilent Technologies 7890B gas chromatograph, equipped with an HP-INNOWAX column (30 m x 0.250 mm x 0.25 µm).

Samples were prepared using the standard addition method, with 25 mg of acetonitrile as internal standard, 125 mg sample, and 850 mg water as solvent. Samples were injected at 0.5 µL volume and a split of 15:1 with an inlet pressure of 12 psi and inlet heater at 250°C.

Table S3: Heating profiles for the analysis of TH reactions using GC-TCD with HP-INNOWAX column.

	Rate [°C min^{-1}]	Temperature [°C]	Holding time [min]
**T_{Start}**		50	2
**Ramp 1**	7	110	0
**Ramp 2**	60	250	55

Table S4: Volume flows via a column for the analysis of hydroformylation using GC-TCD with HP-INNOWAX column.

	Value [ml min^{-1}]
**$n_{constant}$**	1.25

Calibration for the model reaction of levulinic acid (**1**) to γ-valero lactone (**1b**) was performed using a five-point calibration with internal standard and triple measurements:

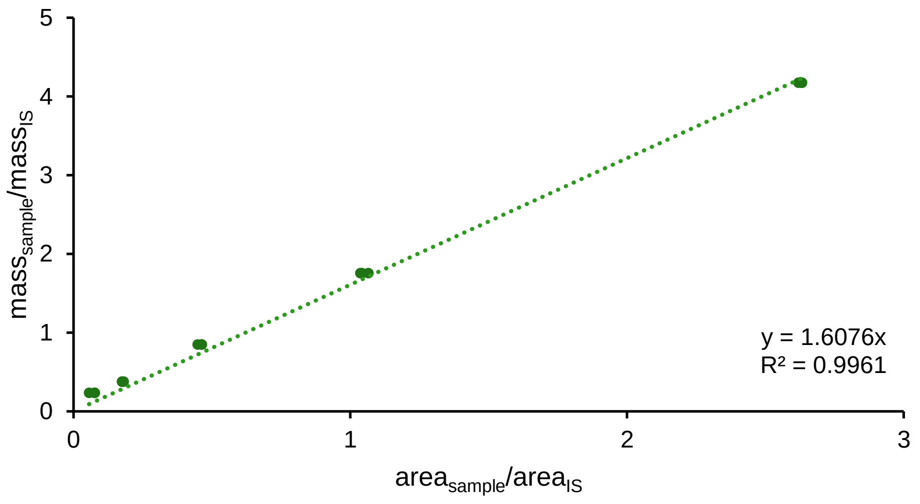

Figure S 4: Calibration curve of levulinic acid (**1**).

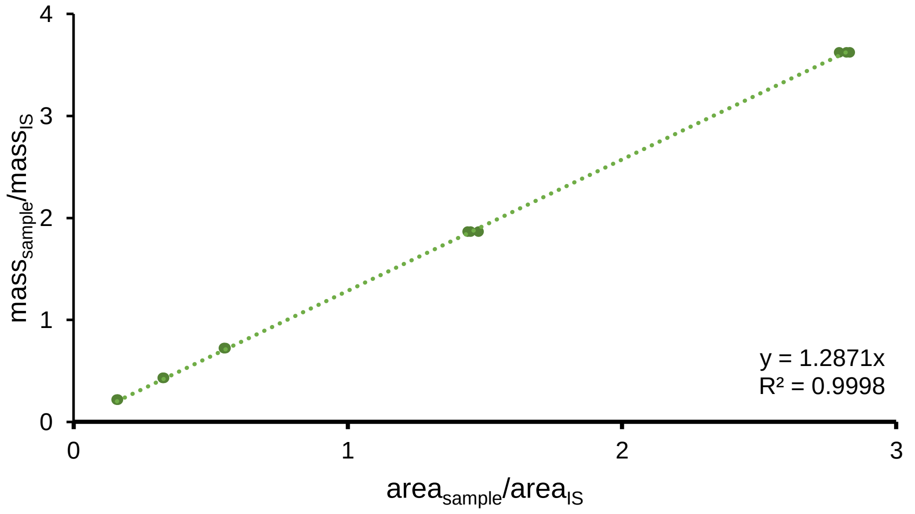

Figure S 5: Calibration curve of GVL (**1b**).

4.3. NMR Analytics

All measurements were performed on Bruker Avance III HD 600 NMR and Bruker Avance Neo 600 NMR spectrometers. Samples were prepared using at least 0.2 mL deuterated solvent for locking and shimming of the magnets. For quantitative experiments, the ^{13}C-inverse gated pulse sequence was employed with an increased D1 to accommodate the slow T_1 relaxation of carbonic acid nuclei.

4.3.1. NMR Spectra Catalyst Synthesis

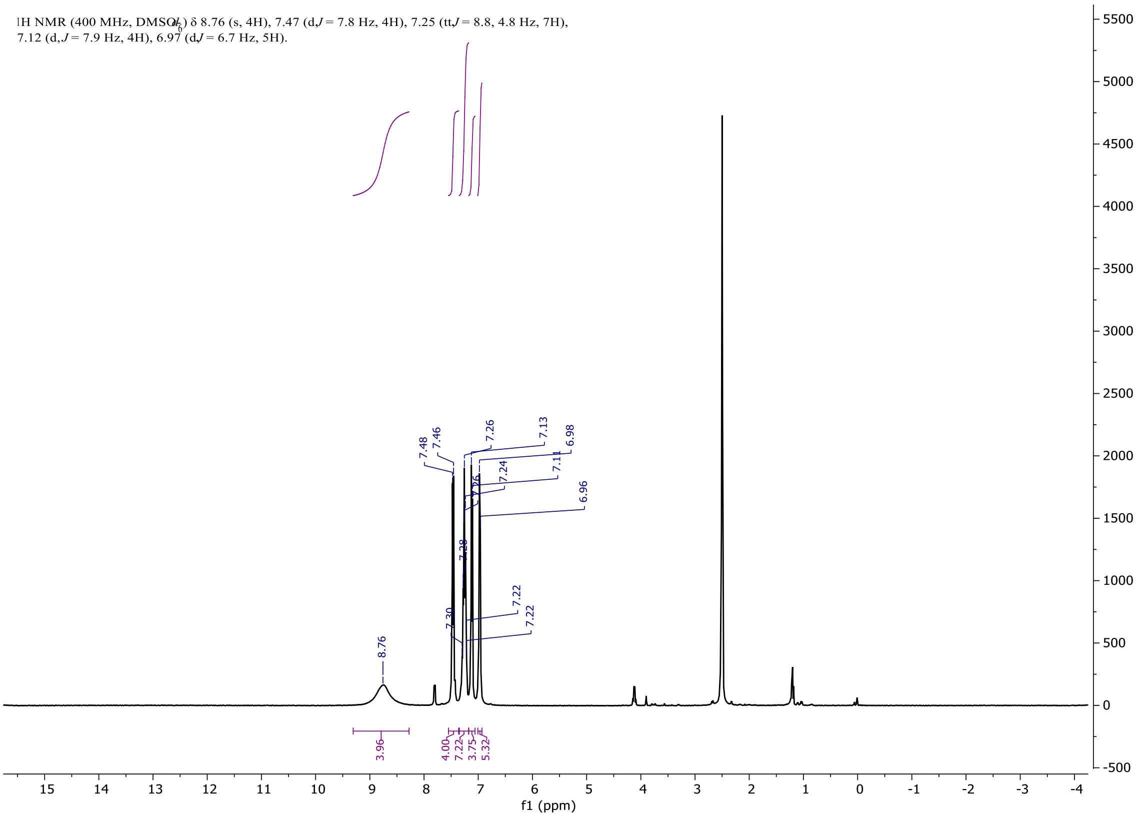

Figure S 6: ^{1}H NMR spectrum (400 MHz) of the disulfonic acid **22**.

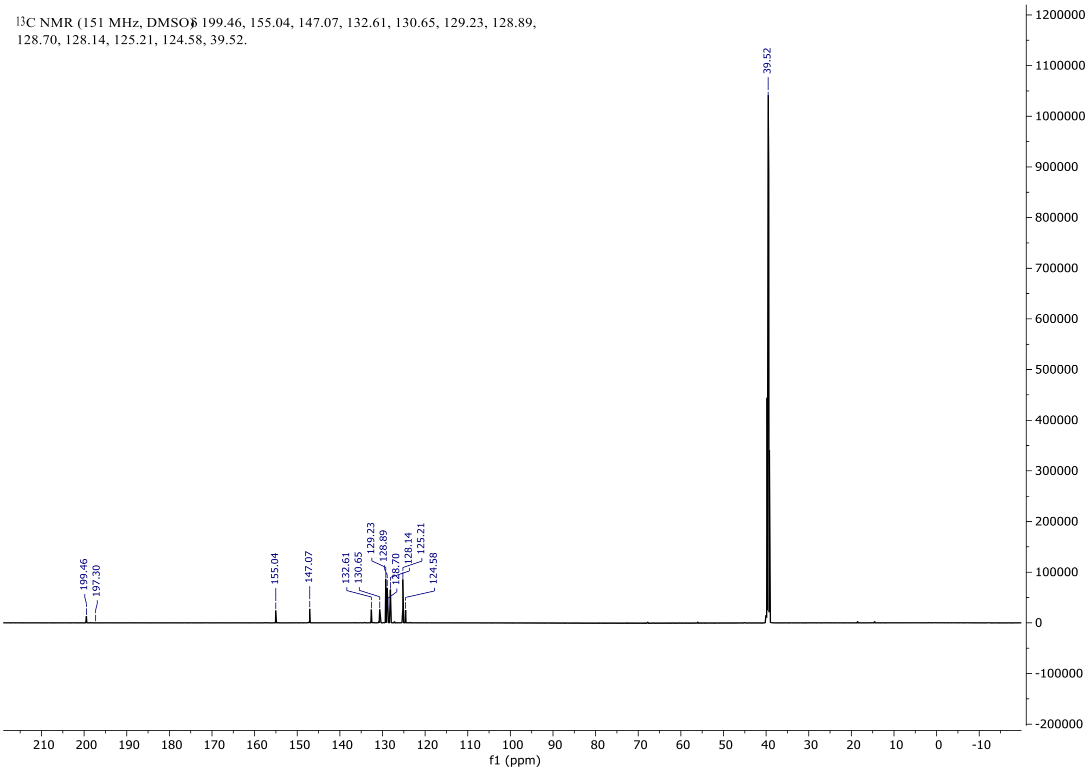

Figure S 7: ^{13}C NMR spectrum (151 MHz) of the disulfonic acid **22**.

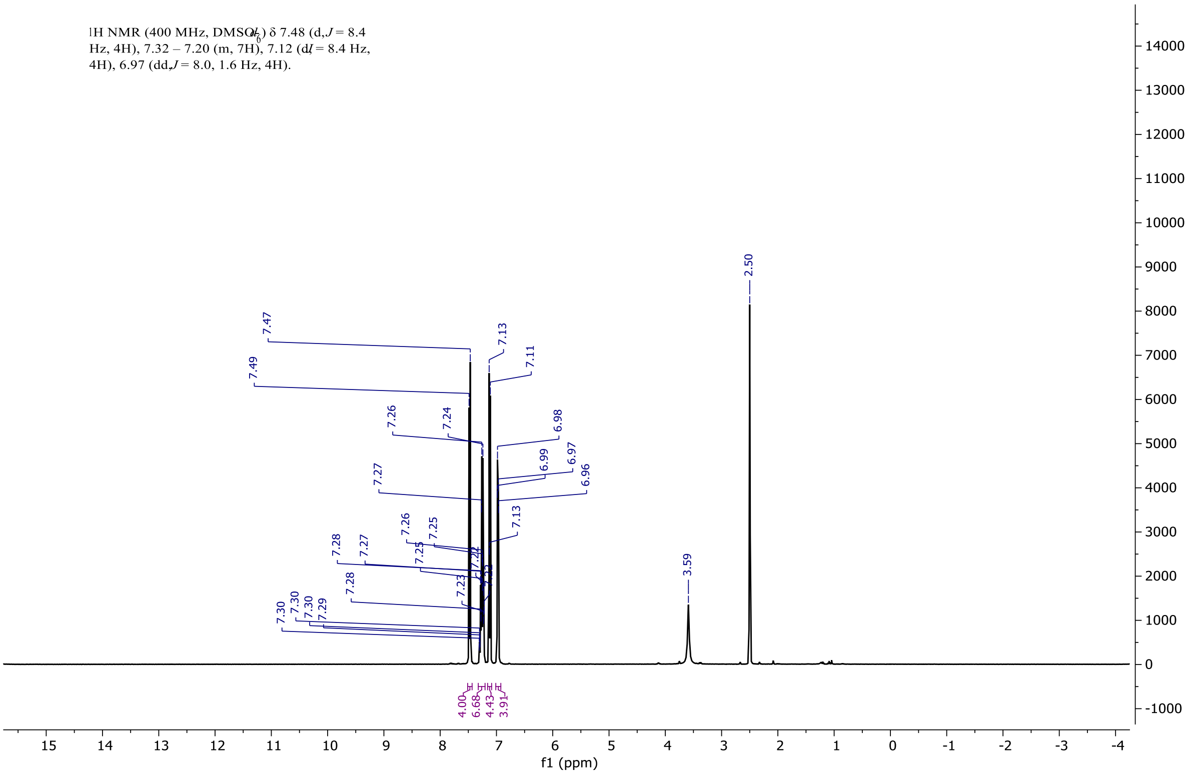

Figure S 8: ^{1}H NMR spectrum (400 MHz) of the disodium sulfonate ligand **23**.

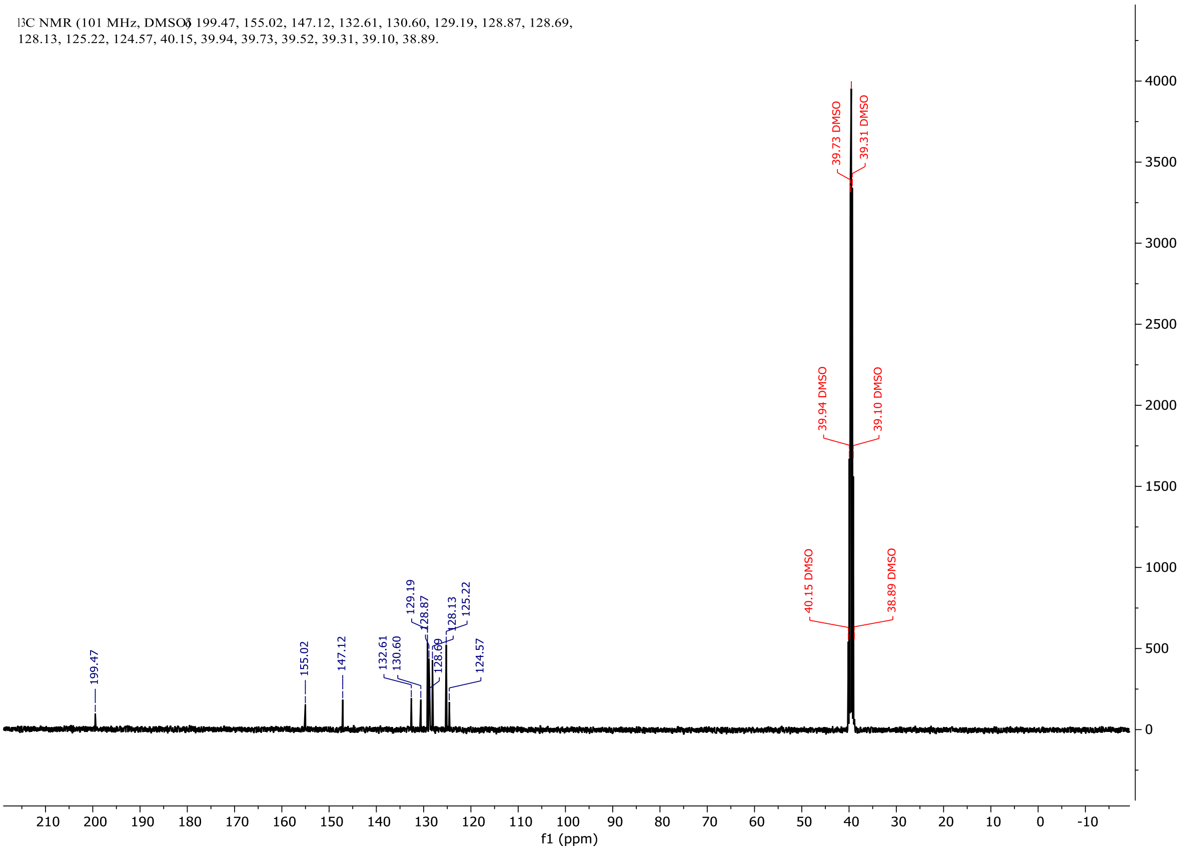

Figure S 9: ^{13}C NMR spectrum (101 MHz) of the disodium sulfonate ligand **23**.

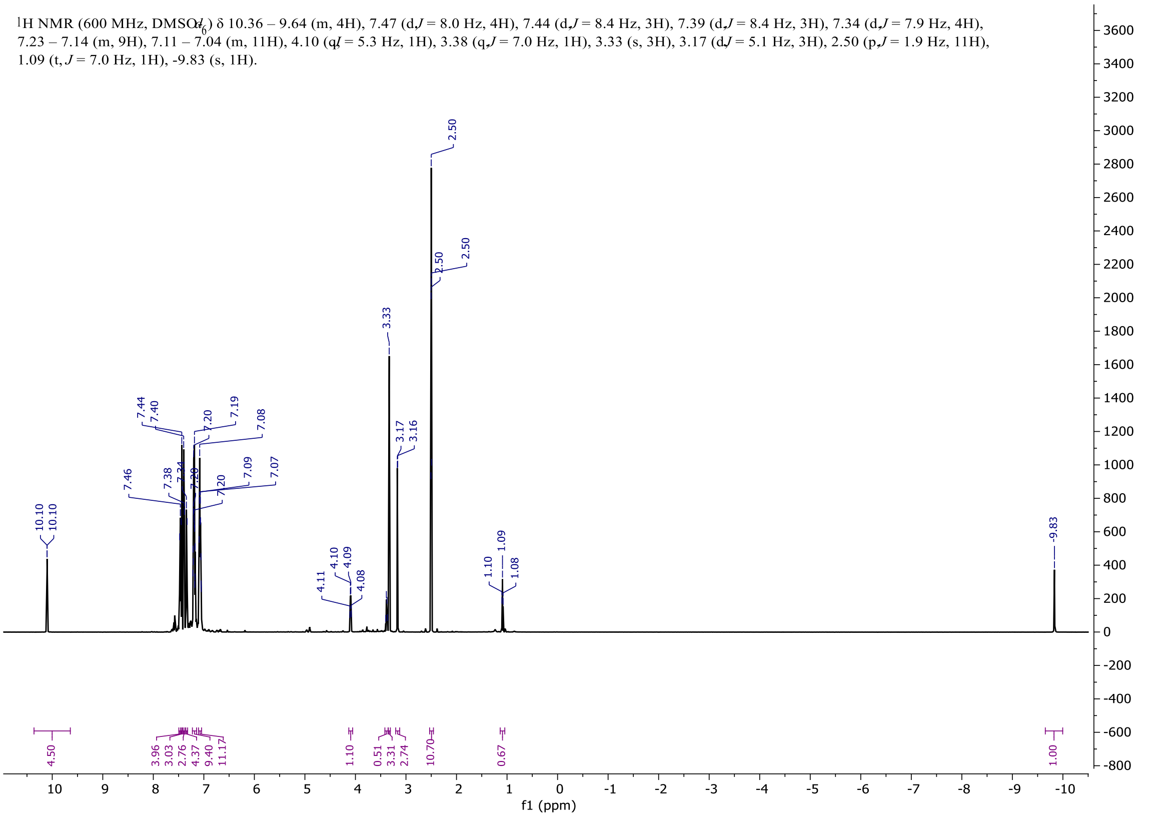

Figure S 10: ^{1}H NMR spectrum (600 MHz) of the orange compound **C1** after 20.5 h (DMSO-d_6, 25 °C).

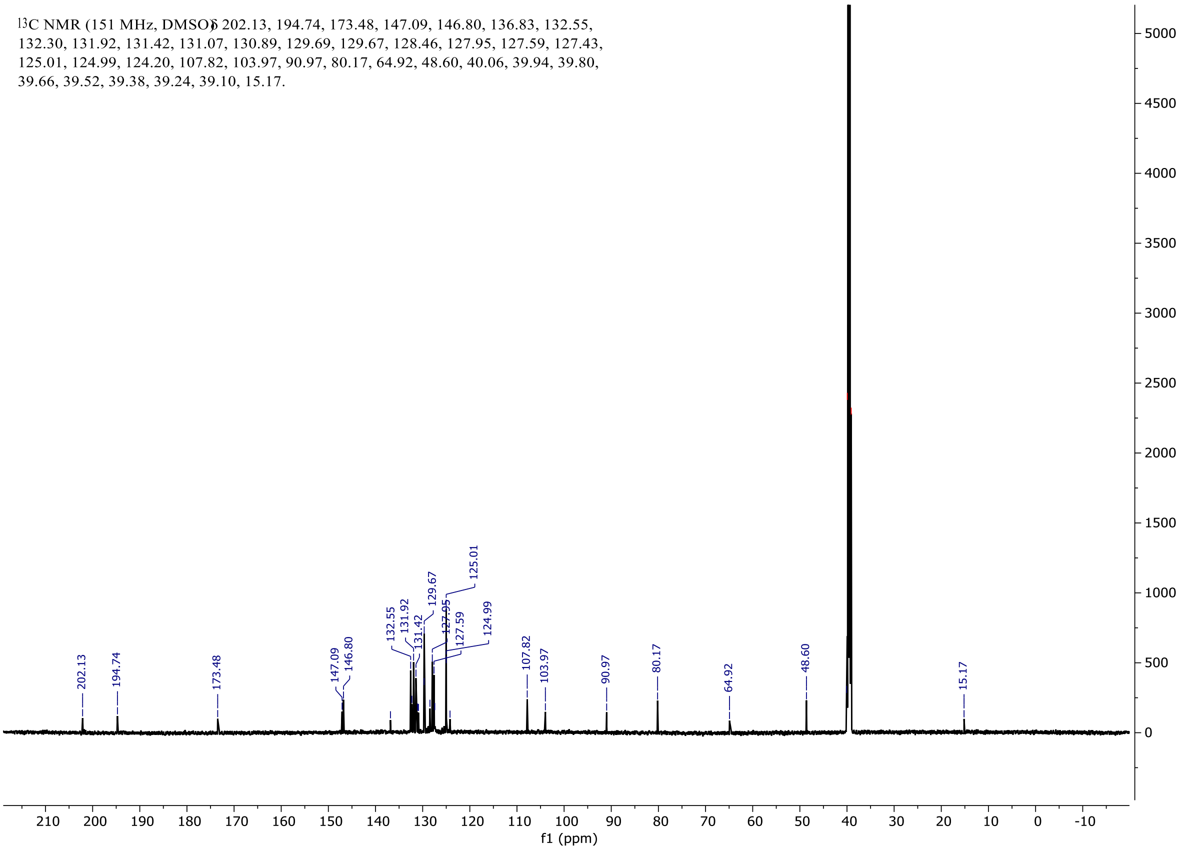

Figure S 11: ^{13}C NMR spectrum (151 MHz) of the orange compound **C1** after 20.5 h (DMSO-d_6, 25 °C).

4.3.2. NMR Spectra Catalyst Deactivation Experiments

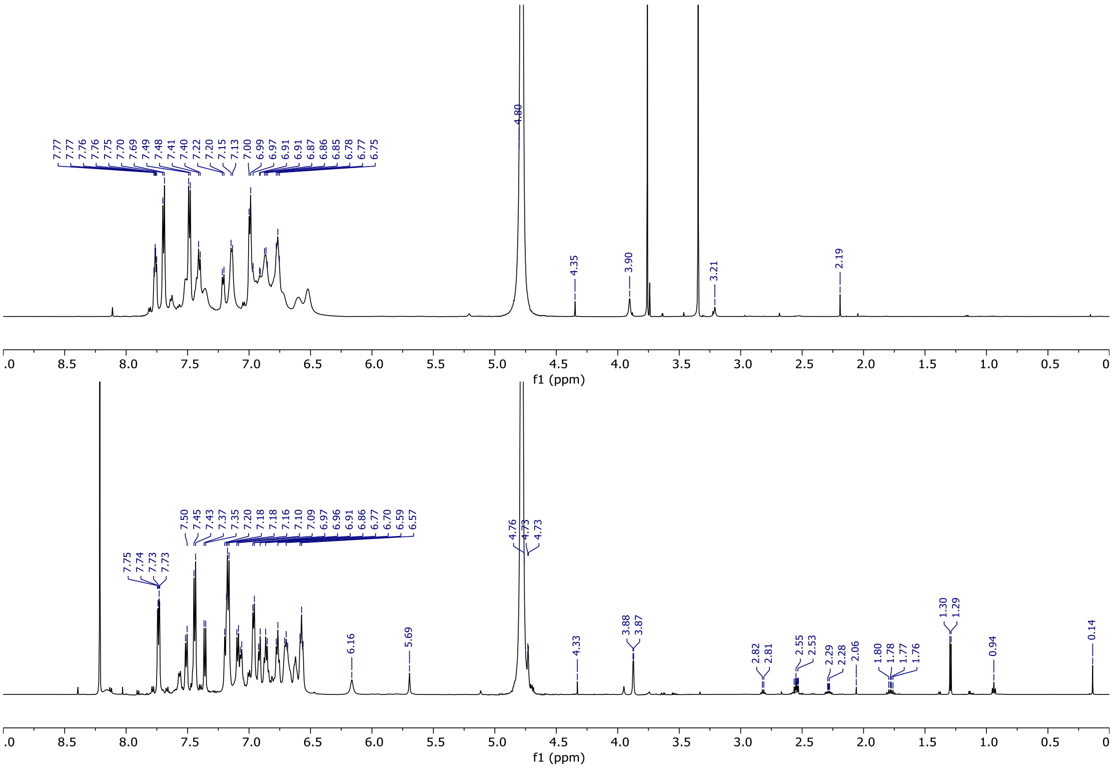

Figure S 12: Stacked ^{1}H NMR spectra (600 MHz) of the catalyst **C1** (top) and **C1** (bottom) heated to 120 °C in an aqueous solution of FA (55 wt%) (D_2O, 25 °C).

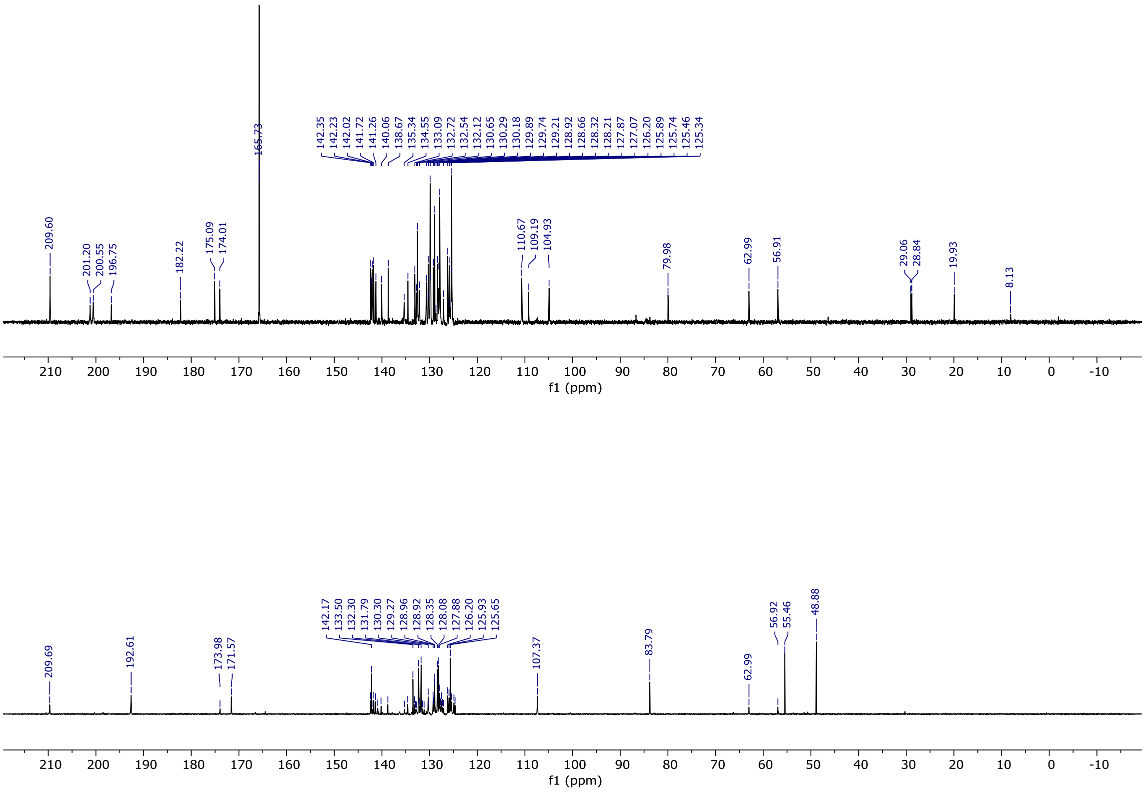

Figure S 13: Stacked ^{13}C NMR spectra (151 MHz) of the catalyst **C1** (bottom) and **C1** (top) heated to 120 °C in an aqueous solution of FA (55 wt%) (D_2O, 25 °C).

4.3.3. Transfer Hydrogenation of 2-Butanone (2)

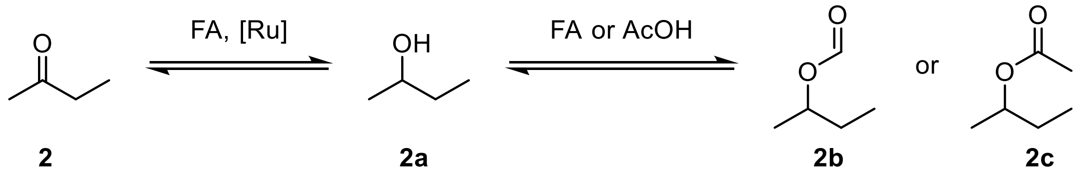

Table S 5: Results of the TH of 2-Butanone (2) based on quantitative ^{13}C-NMR spectroscopy.

Component	x_i [%]
**2**	2
**2a**	13
**2b**	81
**2c**	4

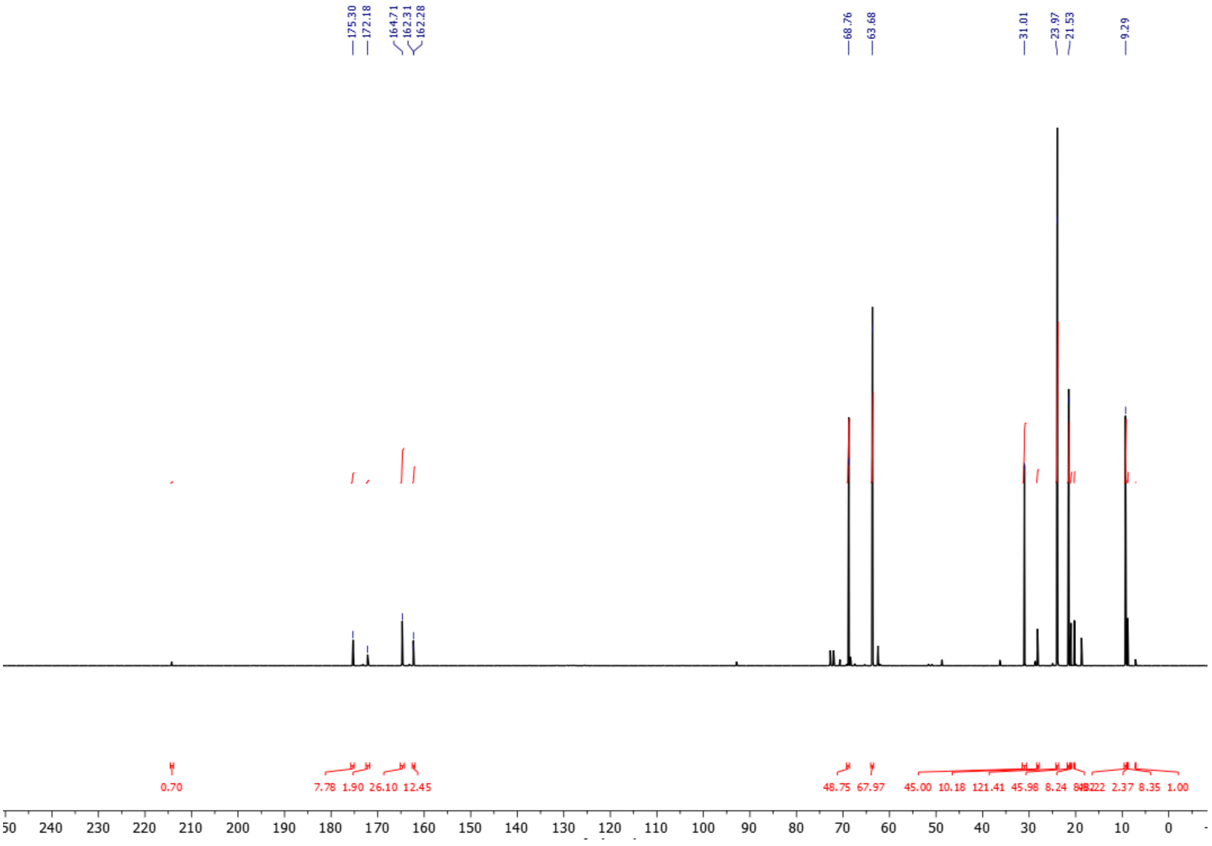

Figure S 14: ^{13}C-NMR (151 MHz, D_2O). TH reaction mixture of 2-butanone (**2**).

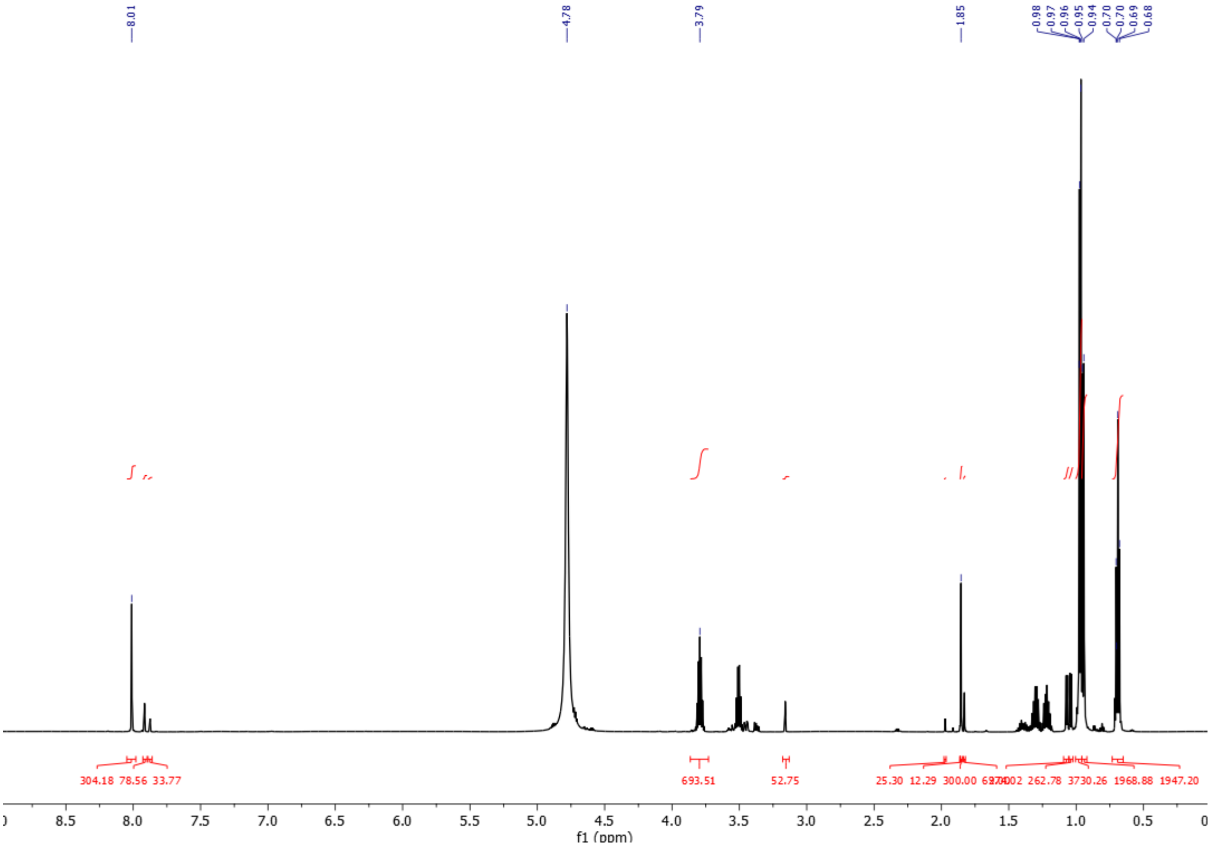

Figure S 15: ^{1}H-NMR (600 MHz, D_2O). TH reaction mixture of 2-butanone (**2**).

4.3.4. Transfer Hydrogenation of 2(5H)-furanone (3)

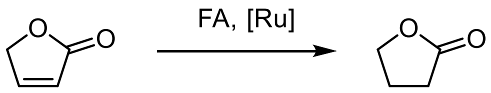

3 **3a**

Table S 6: Results of the TH of 2(5H)-furanone (**3**) based on quantitative ^{13}C-NMR spectroscopy.

Component	x_i [%]
**3**	78
**3a**	22

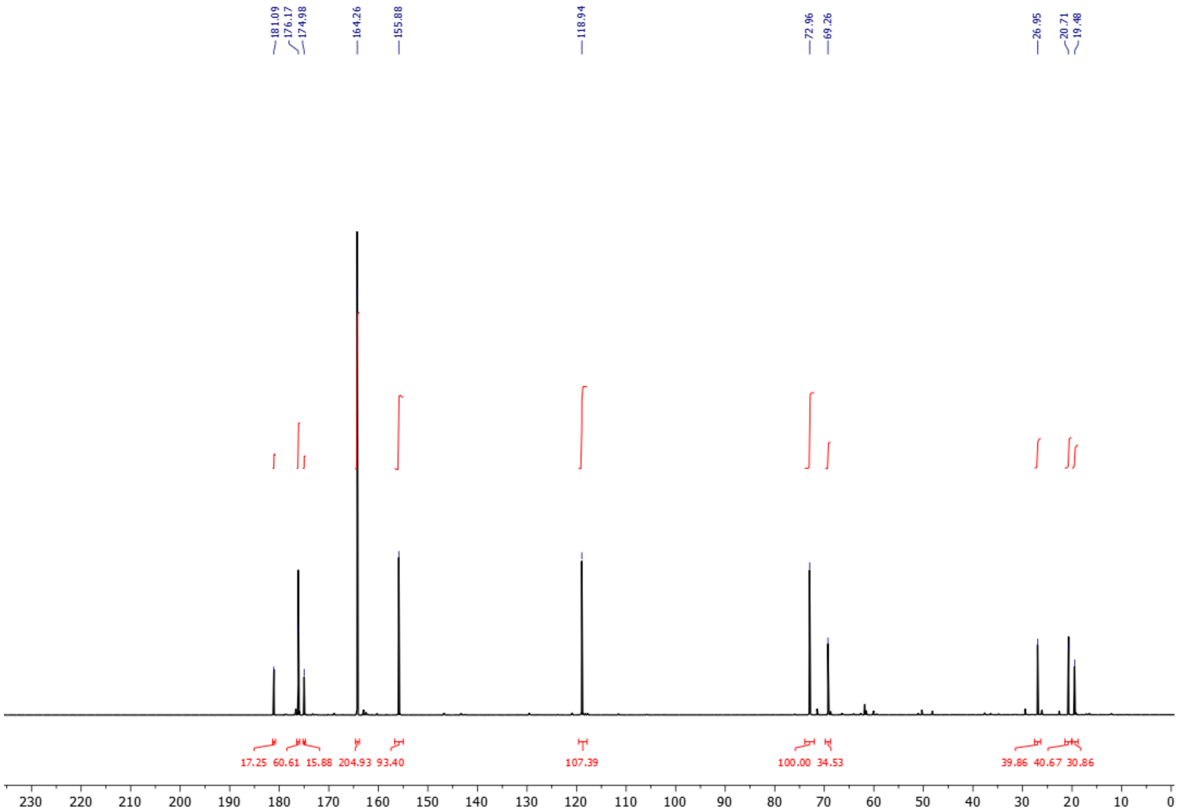

Figure S 16: ^{13}C-NMR (151 MHz, D_2O). TH reaction mixture of 2(5H)-furanone (**3**).

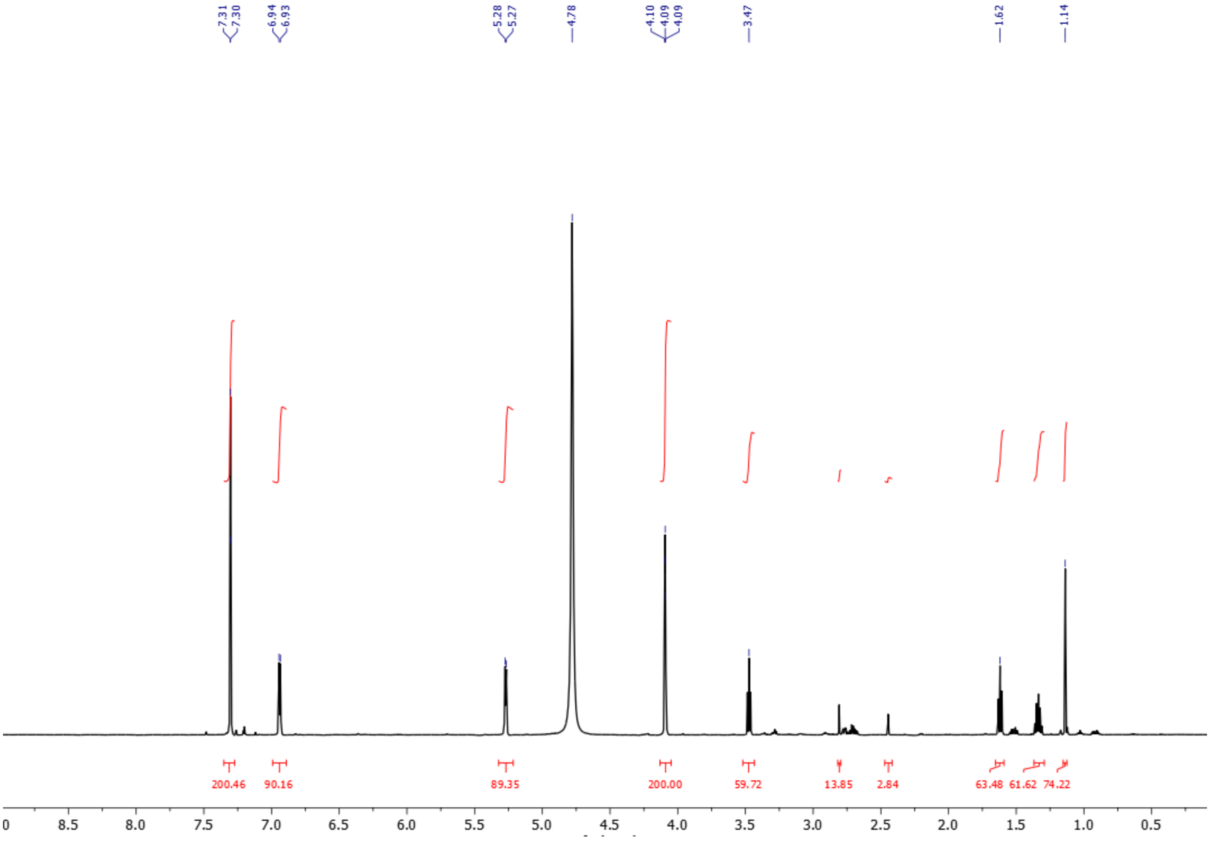

Figure S 17: ^{1}H-NMR (600 MHz, D_2O). TH reaction mixture of 2(5H)-furanone (**3**).

4.3.5. Transfer Hydrogenation of α-Angelica Lactone (4)

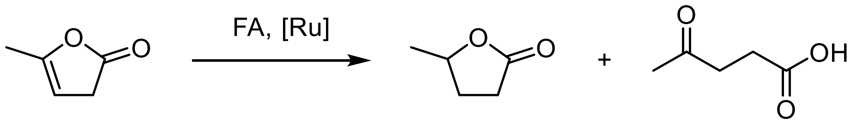

4 **1b** **1**

Table S 7: Results of the TH of α-Angelica Lactone (**4**) based on quantitative ^{13}C-NMR spectroscopy.

**Component**	**x_i [%]**
**4**	0
**1b**	95
**1**	5

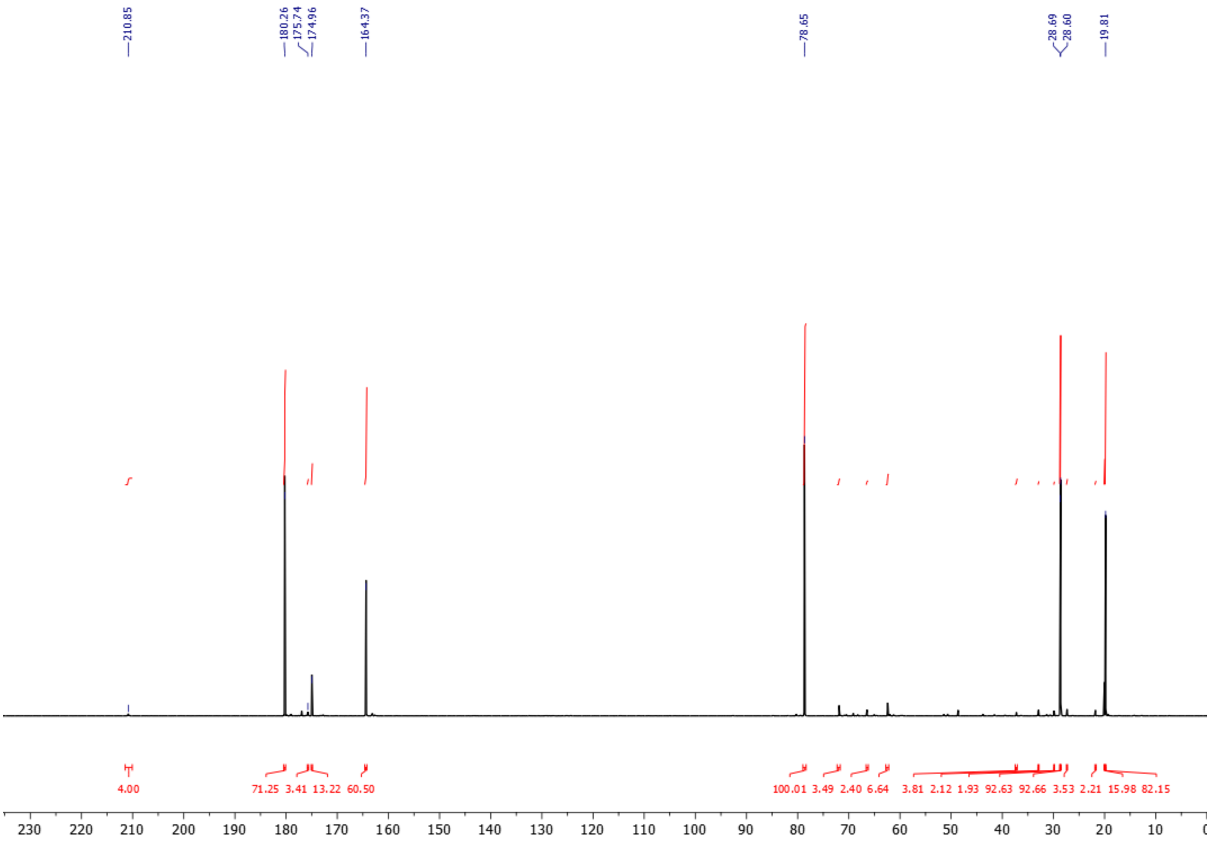

Figure S 18: ^{13}C-NMR (151 MHz, D_2O). TH reaction mixture of α-angelica lactone (**4**).

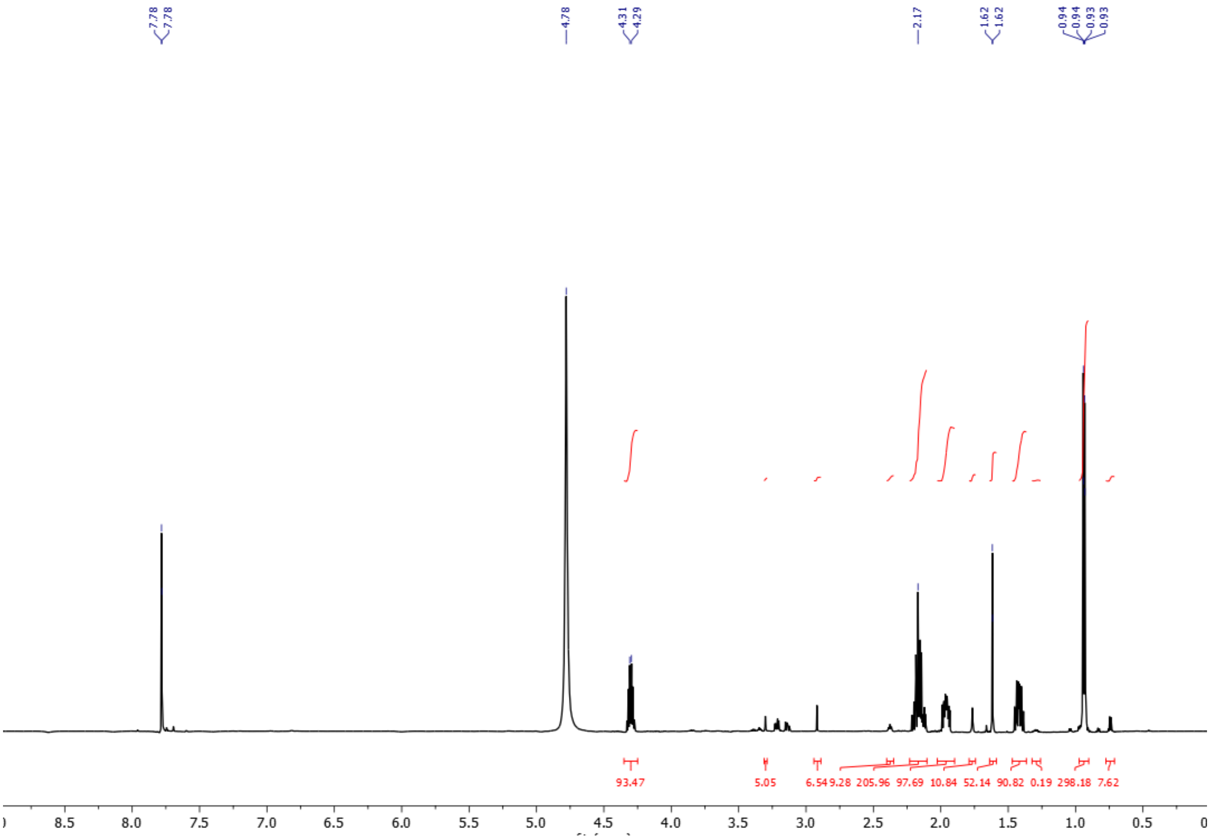

Figure S 19: ^{1}H-NMR (600 MHz, D_2O). TH reaction mixture of α-angelica lactone (**4**).

4.3.6. Transfer Hydrogenation of Dihydroxyacetone (5)

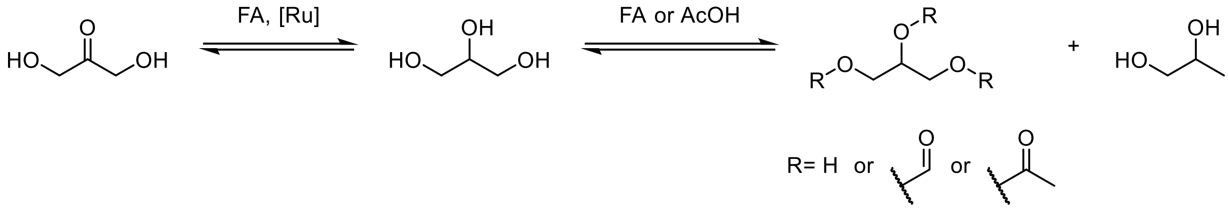

5 **5a** **5b-aa** **5ab**

Table S 8: Results of the TH of dihydroxyacetone (**5**) based on quantitative ^{1}H-NMR spectroscopy.

**Component**	**x_i [%]**
**5**	0
**5a**	78
**5b-aa**	12
**5ab**	10

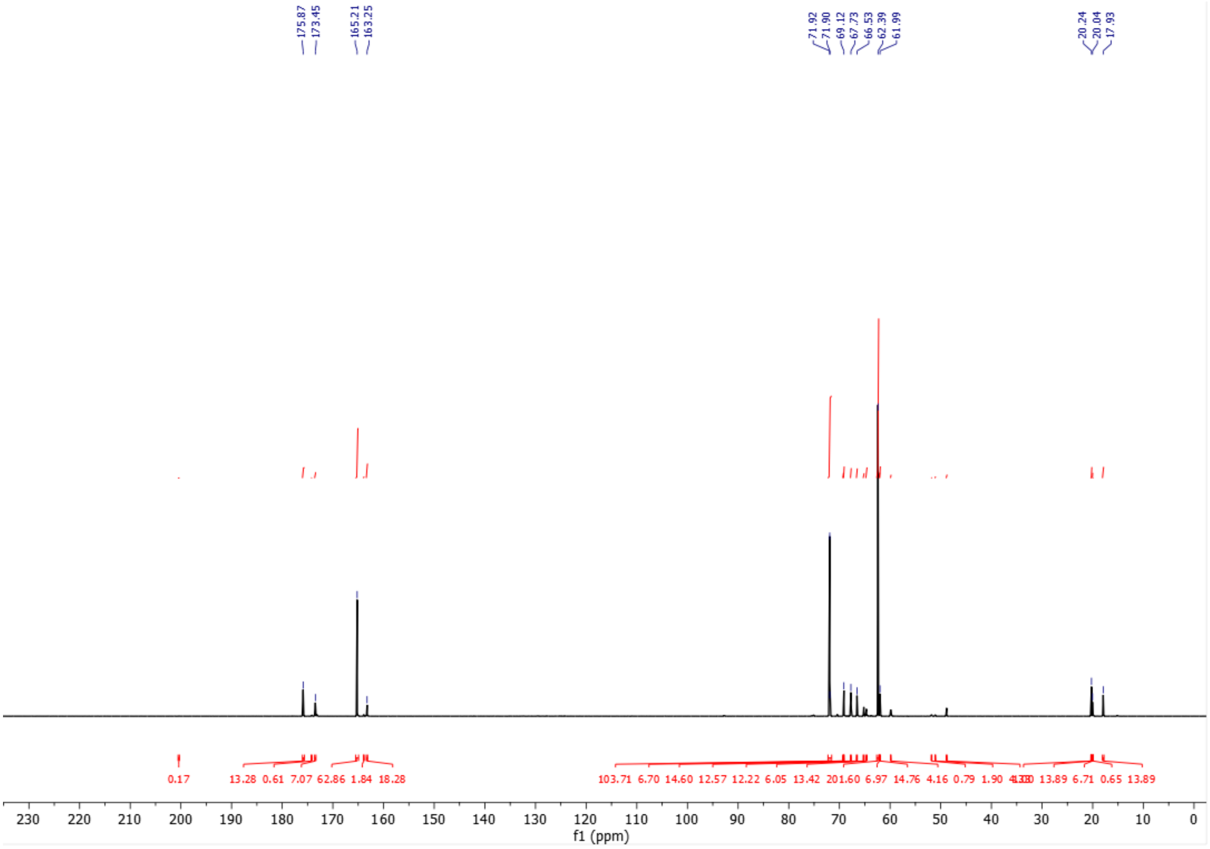

Figure S 20: ^{13}C-NMR (151 MHz, D_2O). TH reaction mixture of dihydroxyacetone (**5**).

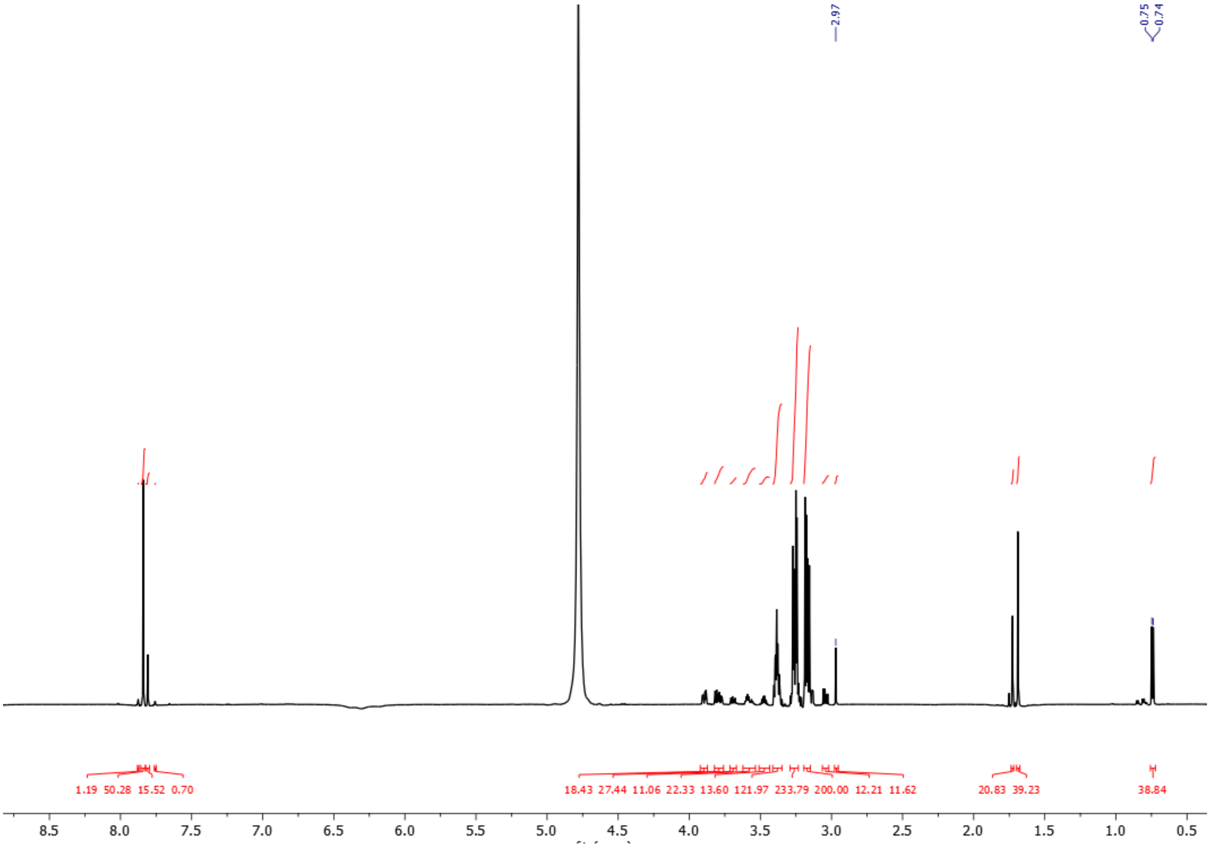

Figure S 21: ^{1}H-NMR (600 MHz, D_2O). TH reaction mixture of dihydroxyacetone (**5**).

4.3.7. Transfer Hydrogenation of Allyl Alcohol (6)

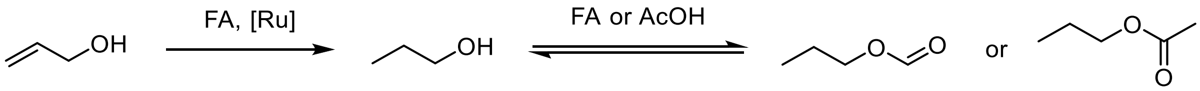

6 6a 6b 6c

Table S 9: Results of the TH of allyl alcohol (**6**) based on quantitative ^{1}H-NMR spectroscopy.

Component	x_i [%]
**6**	0
**6a**	61
**6b**	29
**6c**	10

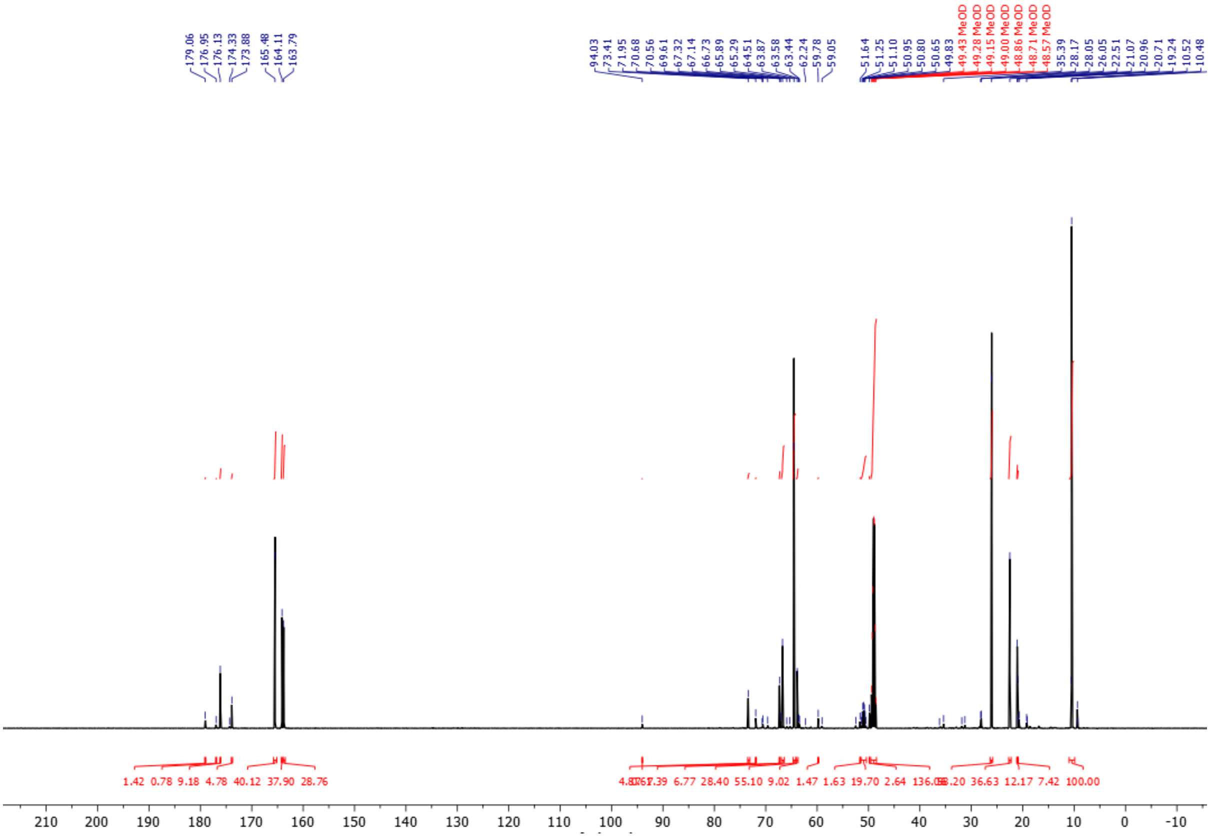

Figure S 22: ^{13}C-NMR (151 MHz, D_2O). TH reaction mixture of allyl alcohol (**6**).

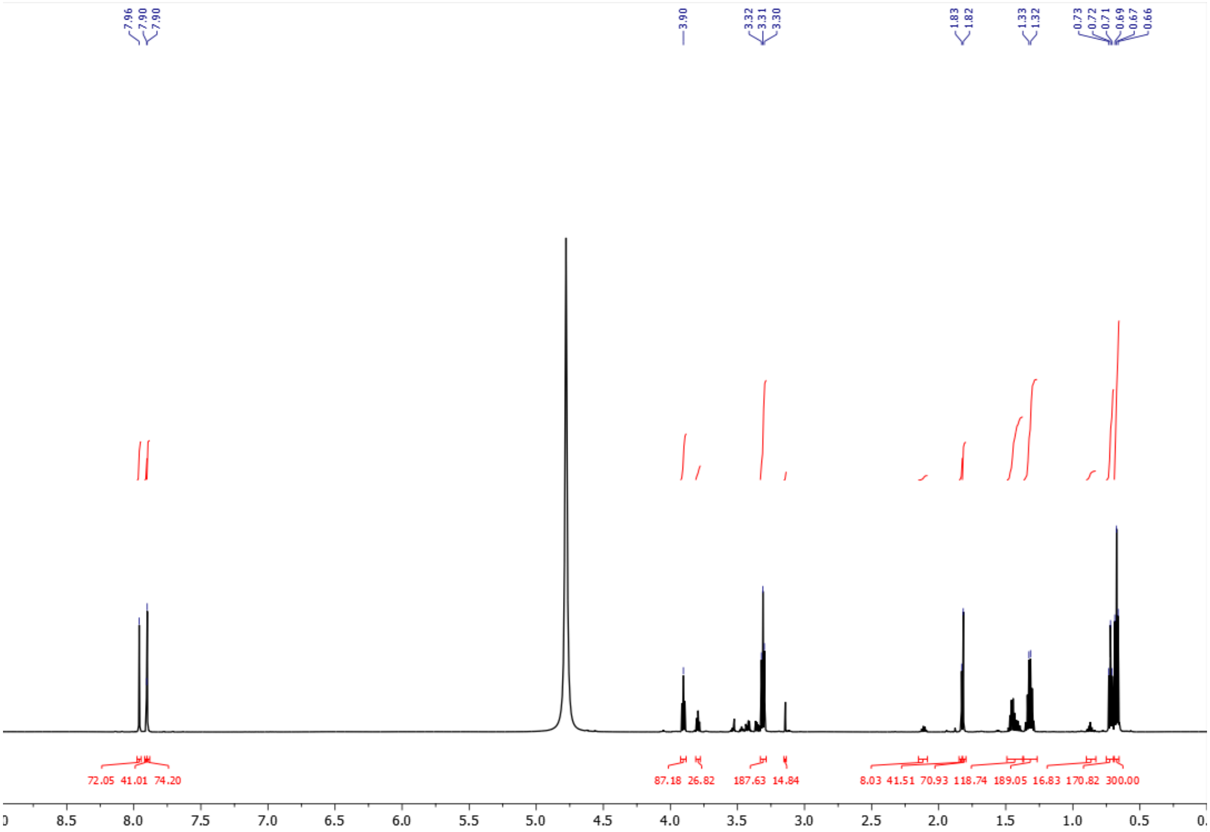

Figure S 23: ^{1}H-NMR (600 MHz, D_2O). TH reaction mixture of allyl alcohol (**6**).

4.3.8. Transfer Hydrogenation of Acetone (7)

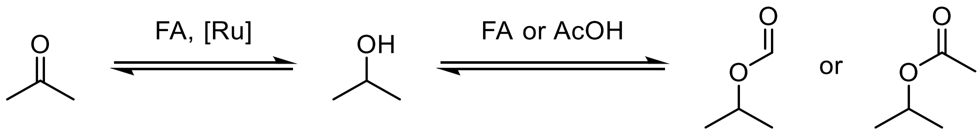

7 **7a** **7b** **7c**

Table S 10: Results of the TH of acetone (**7**) based on quantitative ^{1}H-NMR spectroscopy.

Component	x_i [%]
**7**	2
**7a**	77
**7b**	16
**7c**	5

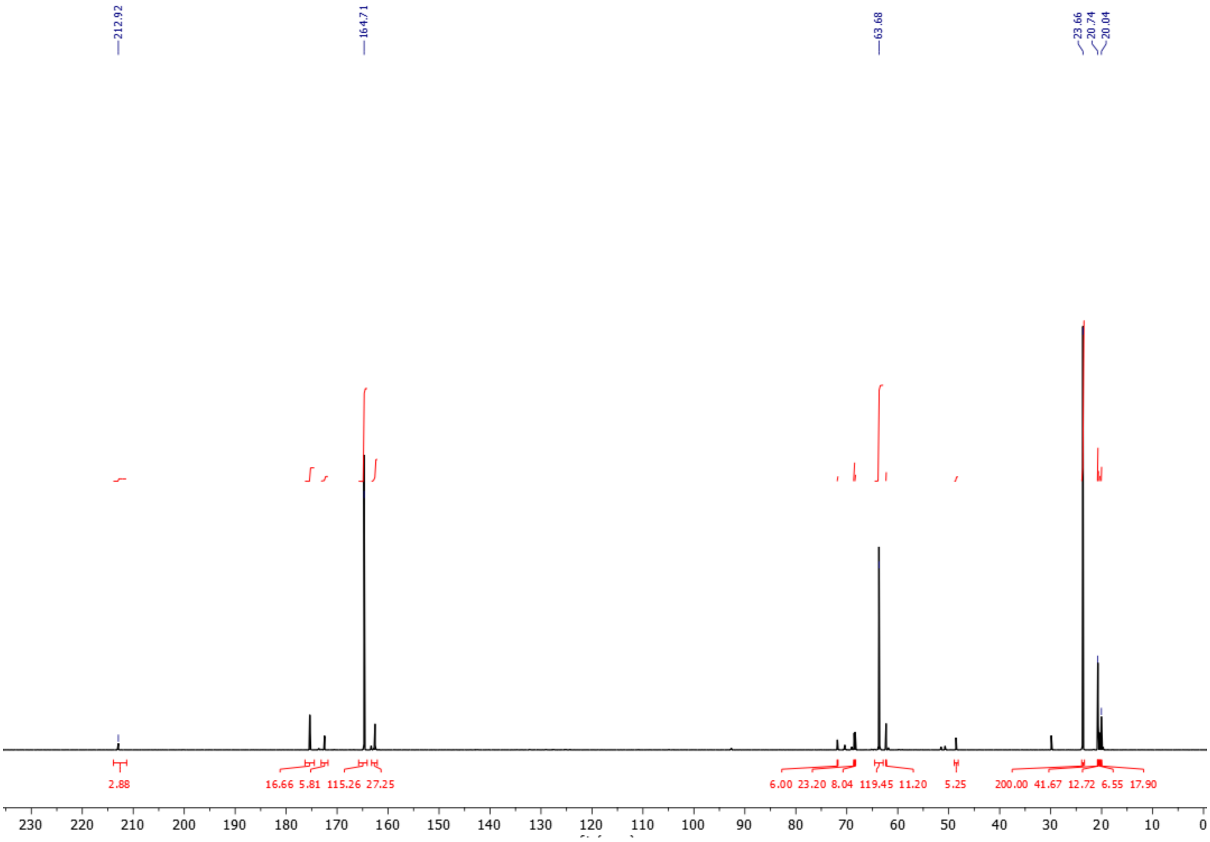

Figure S 24: ^{13}C-NMR (151 MHz, D_2O). TH reaction mixture of acetone (**7**).

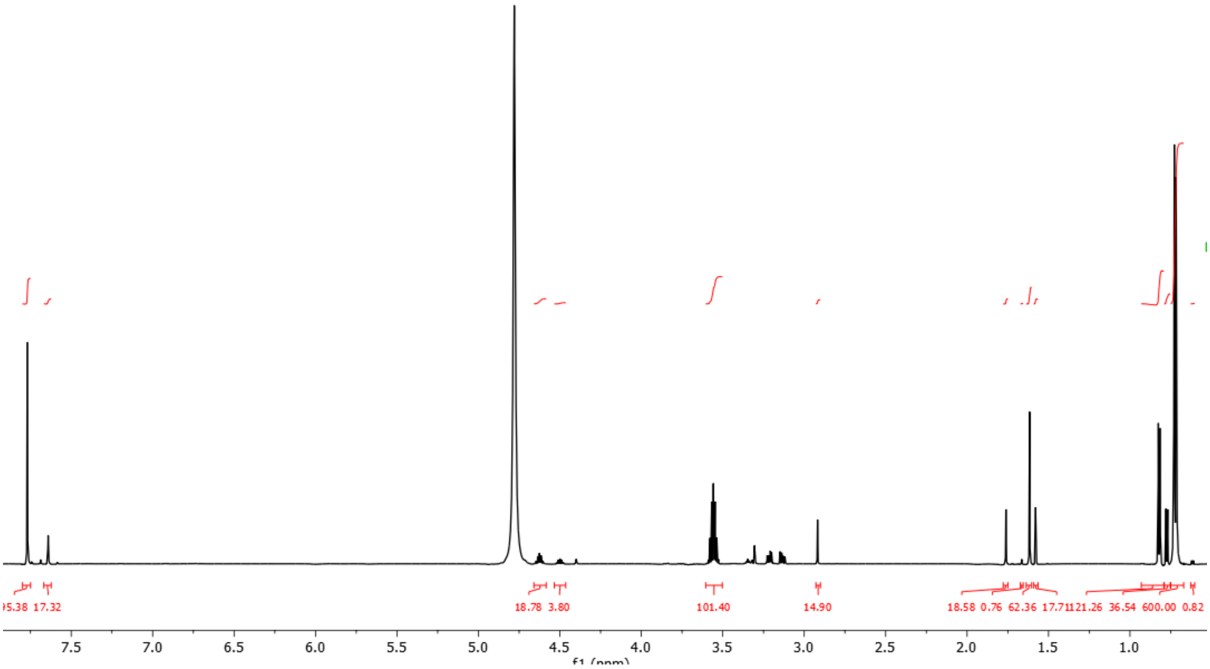

Figure S 25: ^{1}H-NMR (600 MHz, D_2O). TH reaction mixture of acetone (**7**).

4.4. ICP-OES

The elemental composition of the catalyst C1 and the black particles found in the reaction mixture was confirmed by inductively coupled plasma emission spectroscopy (ICP-OES, Analytik Jena (Plasma Quant 9000)).

5. References

[14] J. Diekamp, A. Schmidt, J. J. Holstein, C. Strohmann, T. Seidensticker, "Synthesis and catalytic testing of the first hydrophilic derivative of Shvo's catalyst", *Chem. Commun.*, **2025**, *61*, 117–120.

[19] T. J. G. Skalski, B. Britton, T. J. Peckham, S. Holdcroft, "Structurally-Defined, Sulfo-Phenylated, Oligophenylenes and Polyphenylenes", *J. Am. Chem. Soc.*, **2015**, *137*, 12223–12226.
